# Asperterpenes A and B, two unprecedented meroterpenoids from *Aspergillus terreus* with BACE1 inhibitory activities†
†Electronic supplementary information (ESI) available: 1D and 2D NMR, MS, UV, IR spectra for **1–3**, 1D NMR for **4**. CCDC 1416500 and 1416501. For ESI and crystallographic data in CIF or other electronic format see DOI: 10.1039/c6sc02464e



**DOI:** 10.1039/c6sc02464e

**Published:** 2016-06-27

**Authors:** Changxing Qi, Jian Bao, Jianping Wang, Hucheng Zhu, Yongbo Xue, Xiaochuan Wang, Hua Li, Weiguang Sun, Weixi Gao, Yongji Lai, Jian-Guo Chen, Yonghui Zhang

**Affiliations:** a Hubei Key Laboratory of Natural Medicinal Chemistry and Resource Evaluation , School of Pharmacy , Tongji Medical College , Huazhong University of Science and Technology , Wuhan 430030 , China . Email: zhangyh@mails.tjmu.edu.cn; b School of Basic Medicine , Tongji Medical College , Huazhong University of Science and Technology , Wuhan 430030 , China

## Abstract

Asperterpenes A (**1**) and B (**2**), two 3,5-dimethylorsellinic acid-based meroterpenoids that contain a unique β-oriented Me-21 with an unprecedented 1,2,5-trimethyl-4,9-dioxobicyclo[3.3.1]non-2-ene-3-carboxylic acid moiety, were obtained from *Aspergillus terreus* in very limited amounts of 3.6 mg and 1.8 mg, respectively. The absolute structure of **1** was determined using X-ray diffraction. Because of the low yield of **1**, a comprehensive characterization of the BACE1 inhibitory activities of **1** was completed *via* molecular biological, cell and animal studies guided by *in silico* target confirmation (ISTC). ISTC assays suggested that compounds **1** and **2** might be BACE1 inhibitors. In cell-based tests, asperterpenes A and B, as natural products, exhibited promising inhibitory activities against BACE1, with IC_50_ values of 78 and 59 nM, respectively. LY2811376 (the positive control), one of the most potent clinical BACE1 inhibitors, has shown an IC_50_ value of 260 nM. *In vivo*, compound **1** exhibited activity similar to that of LY2811376 against Alzheimer's disease (AD) in 3xTg AD mice. Taken together, these findings demonstrate that asperterpene A, which contains a novel carbon skeleton, is the first terpenoid to exhibit effective BACE1 inhibitory activity. Moreover, **1** represents a potential lead compound and a versatile scaffold for the development of drugs for the treatment of AD.

## Abstract 2

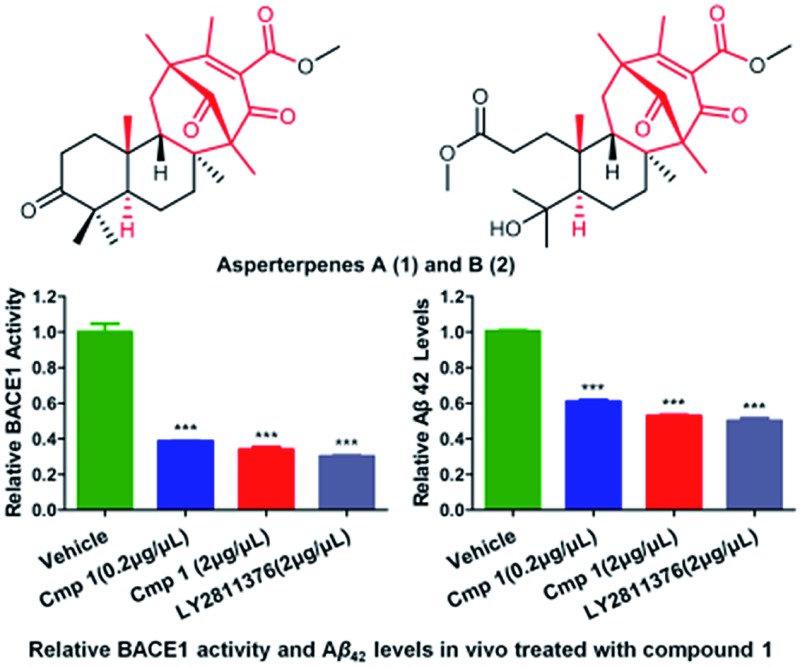
Asperterpenes A and B, two BACE1 inhibitors with unprecedented carbon skeletons isolated from *Aspergillus terreus*, exhibit potent anti-Alzheimer's disease activity.

## Introduction

Alzheimer's disease (AD) is a major, chronic, neurodegenerative illness that is histopathologically characterized by brain β-amyloid (Aβ) plaques and neurofibrillary tangles, which result in a progressive decline in cognitive function and ultimately death.^1,2^ AD is a major public health problem in modern society. Over 30 million people worldwide were affected by this single major cause of dementia in middle-aged and elderly individuals in 2015. By 2050, the number of AD cases could exceed 100 million all over the world.^3^ Drugs such as acetylcholinesterase inhibitors and noncompetitive antagonists of NMDA receptors have been used clinically to alleviate cognitive impairment, but do not target the underlying pathology. New approaches to AD treatment have focused on reducing the production of Aβ, which has been confirmed to be the key contributor to AD by genetic and pathological studies.^4,5^ β-site amyloid precursor protein cleaving enzyme 1 (BACE1, β-secretase) initiates the production of Aβ by cleaving the extracellular domain of the amyloid precursor protein (APP).^6,7^ Consequently, BACE1 is considered a prime therapeutic target, and the development of BACE1 inhibitors is being intensely pursued.^8^ Compared with clinically used drugs, BACE1 inhibitors have direct implications for AD pathology without greatly affecting viability.^9^ In conclusion, there is an urgent demand for the development of BACE1 inhibitors.

**Fig. 1:**
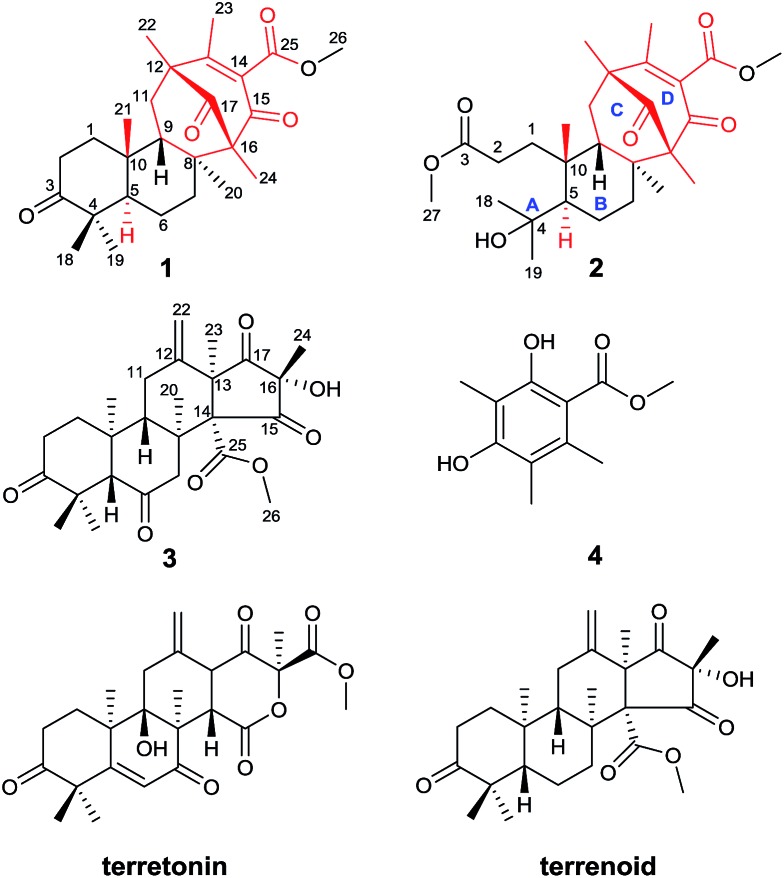
Structures of compounds **1–4** (red: new ring systems and fusion patterns of A/B rings that differ from normal analogues).

As part of our continued investigation of bioactive metabolites from fungi,^10,11^ asperterpenes A (**1**) and B (**2**), two meroterpenoids with unprecedented carbon skeletons, were isolated from the culture broth of *Aspergillus terreus*, along with a new biosynthetically related compound, asperterpene C (**3**) (Fig. 1). Asperterpenes A (**1**) and B (**2**) represent a new class of meroterpenoids, because the ring D is rotated by 180 degrees around the C-12/C-15 axis, which results in an unprecedented 8,16-*cyclo*-8,14-*seco*-6/6/6/6 skeleton that is distinct from those of meroterpenoids such as berkeleytrione and preaustinoid A.^12^ These structural differences lead to a novel carbon skeleton that possesses an unprecedented 1,2,5-trimethyl-4,9-dioxobicyclo[3.3.1]non-2-ene-3-carboxylic acid moiety. In addition, **1** and **2** contain a unique β-oriented Me-21 that differs from previously reported meroterpenoids of 3,5-dimethylorsellinic acid origin, such as berkeleytrione and berkeleyones A–C.^13^ Both **1** and **2** were subjected to *in vitro* experiments guided by *in silico* target confirmation (ISTC) studies and *in vivo* studies were performed for **1**. Compounds **1** and **2** exhibited promising BACE1 inhibitory activities in cell-based assays, with IC_50_ values of 78 and 59 nM, respectively, and were more active than LY2811376 (IC_50_ = 260 nM), a potent clinical BACE1 inhibitor produced by Eli Lilly (Fig. S6†).§
§Crystal data for compound **1**: C_26_H_36_O_5_, *M* = 428.55, orthorhombic, *a* = 7.9305(2) Å, *b* = 11.6311(3) Å, *c* = 25.2575(5) Å, *α* = 90.00°, *β* = 90.00°, *γ* = 90.00°, *V* = 2329.76(10) Å^3^, *T* = 296(2) K, space group *P*2_1_2_1_2_1_, *Z* = 4, *μ*(CuKα) = 0.666 mm^–1^, 9987 reflections measured, 3701 independent reflections (*R*_int_ = 0.0304). The final *R*_1_ values were 0.0354 (*I* > 2*σ*(*I*)). The final w*R*(*F* ^2^) values were 0.1015 (*I* > 2*σ*(*I*)). The final *R*_1_ values were 0.0358 (all data). The final w*R*(*F* ^2^) values were 0.1021 (all data). The goodness of fit on *F* ^2^ was 1.080. Flack parameter = 0.0(2). The Hooft parameter is –0.02(7) for 1531 Bijvoet pairs (CCDC 1416500). Crystal data for compound **3**: C_26_H_36_O_7_, *M* = 474.55, orthorhombic, *a* = 15.9092(4) Å, *b* = 18.9397(5) Å, *c* = 8.0829(2) Å, *α* = 90.00°, *β* = 90.00°, *γ* = 90.00°, *V* = 2435.50(11) Å^3^, *T* = 298(2) K, space group *P*2_1_2_1_2, *Z* = 4, *μ*(CuKα) = 0.770 mm^–1^, 13 869 reflections measured, 3931 independent reflections (*R*_int_ = 0.0479). The final *R*_1_ values were 0.0407 (*I* > 2*σ*(*I*)). The final w*R*(*F* ^2^) values were 0.1136 (*I* > 2*σ*(*I*)). The final *R*_1_ values were 0.0411 (all data). The final w*R*(*F* ^2^) values were 0.1140 (all data). The goodness of fit on *F* ^2^ was 1.046. Flack parameter = 0.14(18). The Hooft parameter is 0.0119(8) for 1939 Bijvoet pairs (CCDC 1416501).
^14^ Moreover, **1** significantly decreased BACE1 activity and Aβ_42_ levels in 3xTg AD mice, similar to LY2811376. These results demonstrate that **1**, with a novel carbon skeleton, is the first terpenoid that acts as an effective BACE1 inhibitor and may be a potential lead compound for the development of AD drugs. Here, we report the isolation, structure elucidation, and plausible biosynthetic pathway of **1** and **2**, and their remarkable ability to inhibit BACE1.

## Results and discussion

The strain of *Aspergillus terreus* was inoculated with solid medium containing 50 kg rice and 50 kg distilled water at 28 °C for 28 days. The growth of fungus was stopped by ethanol after incubation, followed by extraction with ethanol. The EtOH extract of the culture of *A. terreus* underwent repeated silica gel column chromatography, reversed-phase (RP) C_18_ medium pressure liquid chromatography, and Sephadex LH-20 chromatography, followed by semipreparative RP HPLC to afford asperterpenes A (3.6 mg) and B (1.8 mg).

Asperterpene A (**1**) was isolated as colorless cubic crystals. Its molecular formula was determined as C_26_H_36_O_5_ by HRESIMS at *m*/*z* 451.2440 [M + Na]^+^ (calcd 451.2460). The ^1^H and ^13^C NMR data of **1** were partially similar to those of terrenoid, suggesting that **1** is likely to be a terrenoid or terretonin derivative with meroterpenoid features.^15^ The basic carbon skeleton was determined by detailed analyses of the ^1^H-^1^H COSY and HMBC spectra. The ^1^H-^1^H COSY cross-peaks of H-1/H-2 and H-5/H-6/H-7 and HMBC correlations from Me-18 and Me-19 to C-3, C-4 and C-5, from Me-20 to C-7, C-8 and C-9, and from Me-21 to C-1, C-9 and C-10 established the A/B ring system of **1**, which is comparable to that of terrenoids.^15^ In addition to those signals assignable to rings A and B, two carbonyls (*δ*_C_ 198.2 and 210.2), a carboxyl (*δ*_C_ 166.8), an olefinic group (*δ*_C_ 131.3 and 163.9), two quaternary carbons (*δ*_C_ 67.4 and 49.5), a methylene (*δ*_C_ 39.8), three methyls (*δ*_C_ 14.2, 17.7, and 18.6), and a methoxyl (*δ*_C_ 52.5) remained, indicating considerable differences from terrenoids. An α,β-unsaturated carbonyl group (C-13–C-15) was proposed based on the characterized chemical shifts of a carbonyl (*δ*_C_ 198.2) and olefinic group (*δ*_C_ 131.3 and 163.9). This speculation, together with the HMBC correlations from Me-22 to C-12, C-13 and C-17, from Me-23 to C-12, C-13 and C-14, and from Me-24 to C-15, C-16 and C-17, established the presence of the six-membered ring D. Moreover, the ^1^H-^1^H COSY cross-peak of H-9/H-11 and HMBC interactions from Me-20 to C-16, from Me-24 to C-8, and from Me-22 to C-11 revealed that ring D is fused to ring B *via* C-8/C-16 and C-9/C-11/C-12 linkages to form a six-membered ring C. Finally, the HMBC correlation from Me-26 to C-25 suggested the presence of the methyl ester moiety, which was therefore located at C-14 to complete the structure of **1**, considering the chemical shift and quaternary nature of C-14 as well as the molecular formula deduced by HRESIMS. Consequently, the planar structure of compound **1** was determined.

**Fig. 2:**
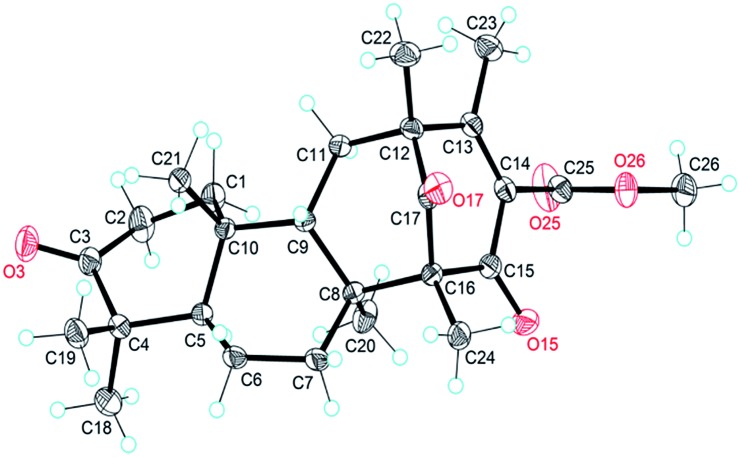
X-ray crystal structure of asperterpene A (**1**).

The NOESY interactions of Me-18/Me-21, H-5/Me-19, H-5/Me-20, and H-9/Me-21 indicated *trans*-fusion patterns for both the A/B and B/C rings. With respect to the conformation of the bi-cyclo[3.3.1]nonane system, the relative configurations of the remaining chiral centers at C-12 and C-16 of **1** should be 12R*,16R* or 12S*,16S*. A NOESY correlation of Me-24/H-7β was clearly observed, consistent with the molecular model of 12R*,16R* and thus establishing the relative configuration of **1**. A single crystal of **1** suitable for X-ray diffraction was obtained by the evaporation of solvent (methanol and chloroform). The X-ray diffraction experiment (Fig. 2) confirmed the structure and the absolute configuration of **1** as 5*R*, 8*S*, 9*S*, 10*R*, 12*R*, 16*R* (for details, see the Experimental section).

Asperterpene B (**2**), obtained as an optically active white gum, possesses the molecular formula C_27_H_40_O_7_ as revealed by the analysis of its HRESIMS data. A comparison of the ^1^H and ^13^C NMR data of **2** with those of **1** suggested that their structures are identical in rings B, C, and D. The main difference between the two compounds was observed in ring A. Detailed analysis of the NMR data revealed that the quaternary carbon at *δ*_C_ 47.0 in **1** was oxygenated in **2** (*δ*_C_ 75.0), and the carbonyl at *δ*_C_ 218.8 in **1** was replaced by a carboxyl (*δ*_C_ 174.6). Moreover, an additional methoxyl was observed at *δ*_C_ 51.7 and *δ*_H_ 3.66. These findings, along with the eight degrees of unsaturation deduced from HRESIMS data, implied that ring A of **2** was open. In the HMBC spectrum, correlations from Me-27 to C-3 and from Me-18 and Me-19 to C-4 and C-5 confirmed the former speculation, and the planar structure of **2** was established.

In the same manner as for **1**, the relative configuration of rings B, C, and D in **2** was confirmed to be identical to **1** using the NOESY spectrum. NOESY interactions of H-1/Me-20 and H-2/H-5 suggested that they are cofacial and α-oriented. In addition, correlations of H-9/Me-21 and H-9/H-7β revealed their β-orientations. Finally, the NOESY correlation Me-24/H-7β implied that the configuration of the bi-cyclo[3.3.1]nonane moiety of **2** was identical to that of **1**. Thus, the relative configurations for all of the chiral centers in **2** were determined to be the same as those of **1**. Considering the shared biosynthetic origin of **1** and **2**, the absolute configuration of **2** was finally determined as 5*R*, 8*S*, 9*S*, 10*R*, 12*R*, 16*R* (Fig. 1).

**Scheme 1:**
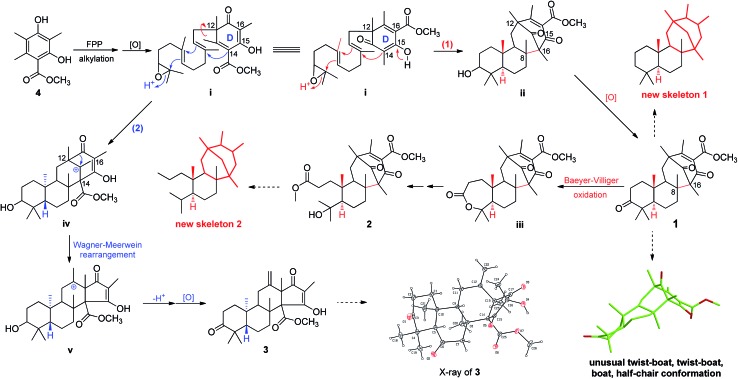
Plausible biosynthetic pathway of **1–3**.

Asperterpenes A (**1**) and B (**2**) possess an intriguing tetracyclic ring system bearing an unprecedented 1,2,5-trimethyl-4,9-dioxobicyclo[3.3.1]non-2-ene-3-carboxylic acid moiety that distinguishes them from known meroterpenoids. A biosynthetic pathway for **1** and **2** was proposed with the co-isolated compound **4** as a precursor (Scheme 1). The biosynthetic pathway of **1** and **2** appears similar to those of the known meroterpenoids with a 6/6/6/6 ring system (such as berkeleytrione, and preaustinoids A and B) and involves a series of cycloadditions and modifications. However, compared to the pathway for known meroterpenoids (from **i** to **iv**, with the key 6/6/6/6-fused tetracyclic intermediate **3**), two main differences are apparent in the pathway for **1** and **2** in step 1. Firstly, although the fusion patterns of the A/B rings in both **ii** and **iv** are *trans*, **ii** bears an unusual β-oriented Me-21. Secondly, due to the rotation of the D ring by 180 degrees around the C-12/C-15 axis, the C-8/C-16 linkage leads to dioxobicyclo[3.3.1]non-2-ene-3-carboxylic acid moieties.^16^ Chair and boat conformations are normally preferred in the cyclohexane of most terpenoids, and thus it is very interesting that **1** has an unusual twist-boat, twist-boat, boat, half-chair conformation, as revealed by its X-ray structure. The position selectivity of C-16 rather than C-14 and the stereoselectivity of Me-21 in the biosynthesis pathway of **1** and **2** are interesting and will attract much attention from the total and biosynthetic chemistry communities.

**Fig. 3:**
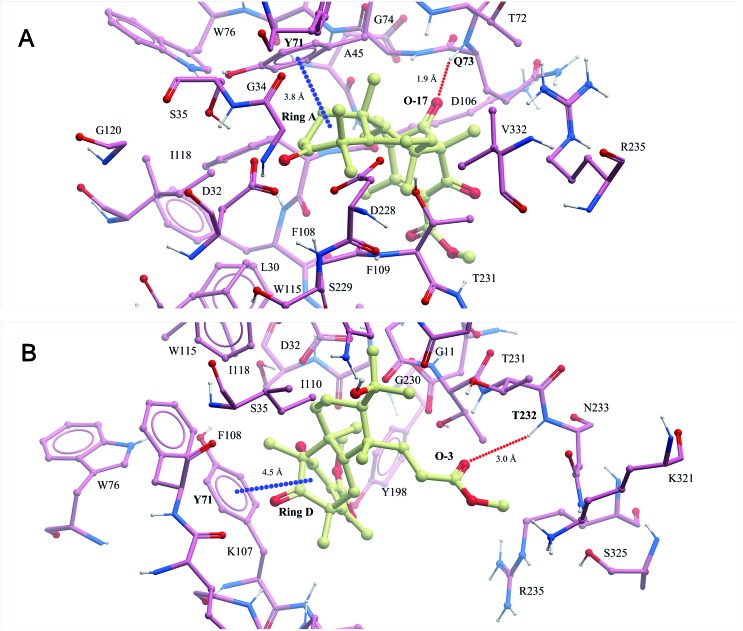
The binding modes of compounds **1** (A) and **2** (B) modelled *in silico* with BACE1 were predicted in the ISTR assays (red dashed lines represent hydrogen bonds and blue dashed lines represent π–π stacking interactions).

In recent years, our group has focused on the investigation of Alzheimer's disease.^17–19^ As a result, asperterpenes A and B were screened against eight significant enzymes *in silico* (PSH, GlpG, KMO, amyloid fibril, APOE4, acetylcholinesterase, NMDA receptor and BACE1) involved in AD processes using ISTR. Targets with lower calculated binding energies are considered to have higher binding affinities for the compounds. Our calculations had sufficient precision to predict that BACE1 exhibited significantly higher binding affinity for **1** and **2** than the other targets (Table S2†). As shown in Fig. 3, O-17 of **1** participates in hydrogen bonding with Q73, and ring A of this compound is involved in a stacking π–π interaction with Y71 in the BACE-1 catalytic pocket. O-3 of **2** participated in hydrogen bonding with T232, and ring D of compound **2** is involved in a stacking π–π interaction with Y71 in the pocket.

**Fig. 4:**
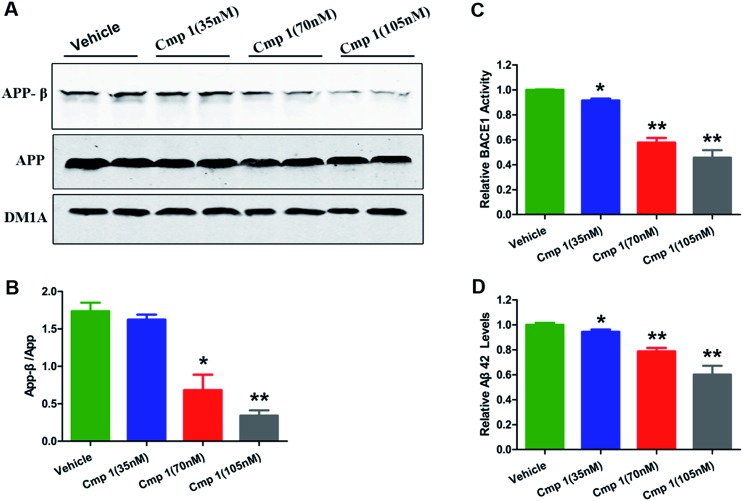
Compound **1** inhibited BACE1 activity and decreased Aβ_42_ production. 35, 70 and 135 nM concentrations of **1** added into the medium of N2a-APP cell lines. (A) Western blot analysis of the protein levels of APP-β, APP and DM1A. (B) Quantitative analysis of the ratio of APP-β/APP in the reference groups. (C) BACE1 activity was determined using β-Secretase Activity Assay Kit. (D) Aβ_42_ levels were quantified through ELISA. Values are shown as mean ± SD. ***p < 0.001, **p < 0.01, *p < 0.05 *versus* the vehicle group.

To confirm our hypothesis, the inhibitory activities of compounds **1** and **2** against BACE1 were further investigated in a cell-based Förster resonance energy transfer (FRET) peptide cleavage assay to validate this mode of action. FRET methods are widely used because they offer a homogeneous and sensitive assay in a multi-well format that is also easily adopted for high-throughput screening (HTS).^20^ **1** and **2** exhibited significant inhibitory effects at the nanomolar scale with IC_50_ values of 78 and 59 nM with the HEK-293 cell line (positive control, LY2811376, IC_50_ = 260 nM), respectively (Table S3†). This investigation suggested that **1** and **2** exhibit greater BACE1 inhibitory activity than LY2811376 in cell-based assays. To further confirm this result, we investigated whether **1** has a similar effect on BACE1 in the N2a-APP cell line.^21^ BACE1 cleaves APP to generate a secreted APP-β fragment and a membrane bound carboxy-terminal fragment (C99). As shown in Fig. 4A and B, β cleavage of APP was significantly decreased in N2a-APP cells treated with **1** at concentrations of 70 and 105 nM, while the levels of full-length APP were unchanged. BACE1 activity and Aβ_42_ were also significantly reduced with these treatments (Fig. 4C and D). These data support the hypothesis that BACE1 activity is significantly inhibited by **1**. In order to evaluate the general toxicity of compounds **1** and **2**, MTT assays were performed, and the exposure of cells to **1** and **2** at concentrations up to 8 μM had no effect on cell viability (Fig. S3†).

**Fig. 5:**
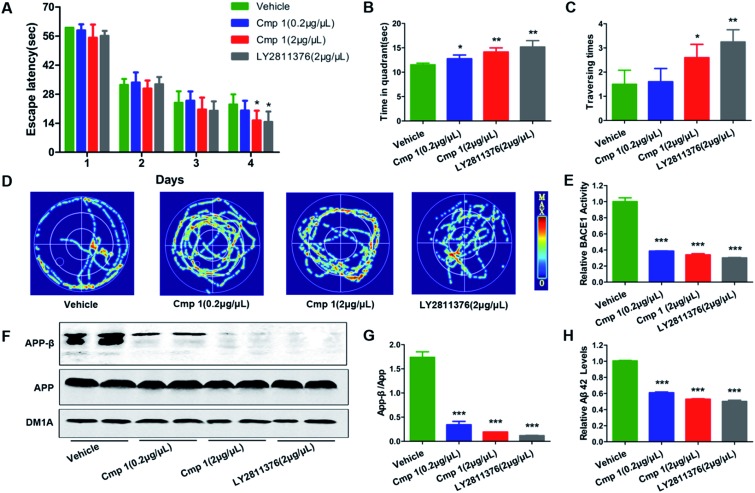
Compound **1** improved learning and memory impairment in 3xTg mice. **1** (2 μg μL^–1^ × 5 μL, 0.2 μg μL^–1^ × 5 μL), LY2811376 (2 μg μL^–1^ × 5 μL) or vehicle was infused into the cerebroventricles of 3xTg mice 48 hours before starting the task. (A) Escape latencies to find the hidden platform were recorded daily. For the memory test, the time spent in the target quadrant (B), the times that the platform was crossed (C) and the swimming tracks (D) were recorded. (F) Western blot analysis of protein levels of APP-β and APP and DM1A, and (G) the quantitative analysis of APP-β/APP. (E) BACE1 activity *in vivo* was determined using β-Secretase Activity Assay Kit. (H) The Aβ_42_ levels *in vivo* were quantified through ELISA. Data are presented as means ± SD. ***p < 0.001, **p < 0.01, *p < 0.05 *versus* the vehicle control (*n* = 10 in each group).

Many natural products exhibit excellent activity *in vitro* but are not effective *in vivo*. To comprehensively evaluate the BACE1 inhibitory activity of **1**, we tested the bioactivity of **1** against AD-like pathological and behavioural alteration using 3xTg mice (the amount of compound **2** was too limited to complete the *in vivo* experiments). The Morris water maze was used to evaluate whether compound **1** could protect against learning and memory impairment in 3xTg mice.^22^ Because hippocampal-dependent memory impairments are the earliest cognitive symptoms detected in AD, we tested all 3xTg mice in a classic spatial reference memory task in the water maze, which relies heavily upon hippocampal integrity (Fig. 5A). We found that only treatment with 2 μg μL^–1^ of **1** or LY2811376 decreased the latency in finding a hidden platform at the 4th day, which partially indicated that compound **1** efficiently attenuated the hippocampal impairment of 3xTg mice. For the probe trial, the crossing numbers and time spent in the target quadrant were significantly increased in mice treated with **1** or LY2811376, which indicated better memory recall (Fig. 5B and C). The typical swimming tracks indicated that mice treated with **1** or LY2811376 searched for the platform in a more appropriate way than the vehicle group, resulting in the shorter latency to locate the platform and more excursions in the target quadrant (Fig. 5D). All of the mice exhibited comparable swimming speeds, indicating that **1** did not affect motor function (Fig. S2†). Thus, compound **1** can protect against learning and memory deficits in the AD mouse model. To test the effect of **1** on BACE1 activity, we employed β-Secretase Activity Assay Kit to detect BACE1 activity in the 3xTg mice.^23^ We found that treatment with **1** dramatically reduced BACE1 activity, similar to treatment with LY2811376 (Fig. 3E), and APP-β/APP was also dramatically decreased, which suggested that BACE1 activity was inhibited (Fig. 5F and G). As BACE1 contributes to the production of Aβ by cleaving APP, we also found a marked decrease in Aβ_42_ production in the hippocampus of 3xTg mice treated with compound **1** (Fig. 5H), while Nissl staining revealed no reduction in cell numbers in the hippocampus (Fig. S1†).

**Fig. 6:**
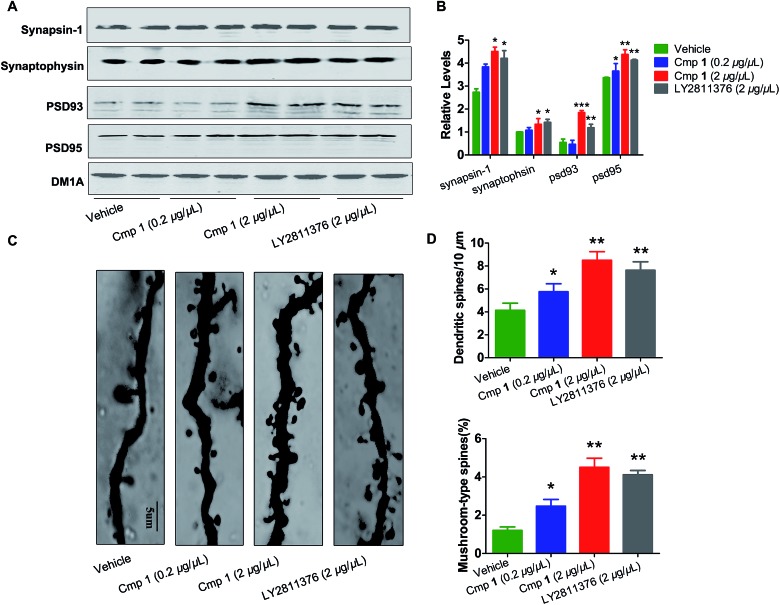
Compound **1** attenuated synaptic toxicity in the hippocampus of 3xTg mice. (A) Western blot analysis of the protein levels of synapsin-1, synaptophysin, psd 95 and psd 93, and their (B) quantitative analysis. (C) Representative photomicrographs of primary dendrites in the hippocampal CA3 region. (D) Quantification of dendrites and mushroom-type dendrites. Values are shown as mean ± SD ***p < 0.001, **p < 0.01, *p < 0.05 *versus* the vehicle group alone (*n* = 10 in each group).

The morphological structures of neurons and the expression of synaptic proteins are critical components for learning and memory.^24,25^ Therefore, we explored the mechanism underlying the improvement in memory induced by compound **1**. Golgi staining demonstrated that treatment with compound **1** remarkably increased dendritic branches and mushroom-type spines in the hippocampus (Fig. 6C and D). We also observed that the expression of several key learning- and memory-related proteins, synapsin-1, synaptophysin, PSD 93 and PSD 95, were increased in compound **1**-treated 3xTg mice (Fig. 6A and B). Overall, **1** effectively improved Aβ-induced synapse impairments *in vivo*, producing effects similar to those elicited by treatment with LY2811376.

The yield of novel, natural isolates is typically too low to perform comprehensive biological assessments, particularly in animals, which severely restricts the discovery of new drugs and hampers the development of modern medicine. In this study, we only obtained 3.6 mg of compound **1** and 1.8 mg of **2** in total and with titres of 0.036 mg kg^–1^ and 0.018 mg kg^–1^, respectively. To compensate for the limited amounts of these compounds, a series of effective methods were used to evaluate BACE1 inhibitory activity guided by ISTC assays. Overall, these methods represent a novel starting point in the discovery of BACE1 inhibitors and may facilitate compound activity screening in medicinal chemistry.

## Conclusions

In this study, asperterpenes A (**1**) and B (**2**), the first natural products with an unprecedented carbon skeleton featuring a 1,2,5-trimethyl-4,9-dioxobicyclo[3.3.1]non-2-ene-3-carboxylic acid moiety, were obtained from *Aspergillus terreus*. We subjected novel compounds **1** and **2** to anti-AD assays based on our previous study and determined that BACE1 was the most likely target guided by the method of ISTC. Asperterpenes A and B displayed significant inhibitory activity against BACE1 *in vitro* and were determined to be the most effective natural BACE1 inhibitors, with even higher BACE1 inhibitory activity than one of the most potent potential clinical BACE1 inhibitors, LY2811376, from Eli Lilly. Many BACE1 inhibitors have strong activity *in vitro* but no effect *in vivo*. However, compound **1** displayed excellent BACE1 inhibitory activity in animal studies, comparable to that of LY2811376. Overall, this is the first report of terpenoids as effective inhibitors of BACE1. Inhibitors of BACE1 are being intensely pursued, and these findings may provide a lead compound for promising drug candidates for the treatment of AD.

## Experimental section

### General experimental procedures

HRESIMS data were obtained in the positive ion mode on a Thermo Fisher LC-LTQ-Orbitrap XL spectrometer. Extensive NMR spectra were obtained on a Bruker AM-600/400 spectrometer with tetramethylsilane as an internal standard, and the ^1^H and ^13^C NMR chemical shifts were normalized to the solvent or solvent impurity peaks for CDCl_3_ at *δ*_H_ 7.24 and *δ*_C_ 77.23. UV and FT-IR spectra were measured using Varian Cary 50 and Bruker Vertex 70 devices, respectively. Optical rotations were recorded using a Perkin-Elmer 341 polarimeter. Melting points were determined *via* a Beijing Tech X-5 micro-melting point instrument (uncorrected). Experimental ECD data were acquired through a JASCO J-810 spectrometer (Jasco, Easton, MD, USA). The X-ray diffraction experiments were carried out on a Bruker APEX DUO diffractometer using graphite monochromated CuKα radiation (*λ* = 1.54178 Å). HPLC procedures were implemented on an Agilent 1200 quaternary system with a UV detector using a reversed-phased C_18_ column (5 μm, 10 × 250 mm, Welch Materials, Inc.) at a flow rate of 2.0 mL min^–1^. CHIRALPAK®IC column (5 μm, 4.6 × 250 mm, Daicel Chiral Technologies Co., Ltd. China) was applied to the enantioseparation. Thin-layer chromatography (TLC) was performed with RP-C_18_ F254 plates (Merck, Germany) and silica gel 60 F254 (Yantai Chemical Industry Research Institute). ODS (50 μm, YMC Co. Ltd., Japan), Sephadex LH-20 (GE Healthcare Bio-Sciences AB, Sweden), and silica gel (200–300 mesh; Qingdao Marine Chemical Inc., China) were used for column chromatography.

### Fungal material

The fungus *Aspergillus terreus* was isolated from the soil collected in the bottom of Yangzi River in May 2013 at Tongji Medical College, Hubei Province, China. The sequence data for this strain have been submitted to the DDBJ/EMBL/GenBank under accession no. KT360948. A voucher sample, QCX20130513, was preserved in the herbarium of Huazhong University of Science and Technology, P. R. China.

### Fermentation and isolation

To prepare the seed culture, the strain was cultured on potato dextrose agar (PDA) at 28 °C for 7 days. The agar plugs were cut into small pieces (approximately 0.6 × 0.6 × 0.6 cm^3^), then the strain was inoculated into 50 Erlenmeyer flasks (5 L) that were previously sterilized by autoclaving and each contained 1000 g rice and 1000 mL distilled water. The flasks were incubated at 28 °C for 28 days. After the incubation, the growth of fungus was stopped by adding 1000 mL ethanol to each flask, followed by extraction with ethanol. We used reduced pressure to remove the ethanol and yield a brown extract (880.0 g). The extracts were introduced to a silica gel chromatography column (CC) and eluted with CH_2_Cl_2_/MeOH (10 : 1–1 : 1) to obtain six fractions (Fr. 1–Fr. 6). We separated Fr. 4 with repeated silica gel CC to yield seven subfractions (Fr. 4.1–Fr. 4.7), and then subjected subfraction Fr. 4.4 to a Sephadex LH-20 CC (CHCl_3_–MeOH, 1 : 1) to afford four parts (Fr. 4.4a–Fr. 4.4d). The second part (Fr. 4.4b) was chromatographed on ODS (MeOH–H_2_O, 20–100%) to yield five mixtures (A–E). Mixture B was purified by repeated semi-preparative HPLC (MeOH–H_2_O, 60% and MeCN–H_2_O, 45%) to yield **2** (1.8 mg). Purifying mixture D by repeated semi-preparative HPLC (MeCN–H_2_O, 65% and MeOH–H_2_O, 55%) yielded **1** (3.6 mg). Finally, the mixture C was separated by semi-preparative HPLC (MeCN–H_2_O, 70%) to get **3** (3.1 mg) and **4** (3.2 mg).

#### Compound **1**

Colorless crystals, [*α*]20D +47.7 (*c* 0.9, MeOH); UV (MeOH) *λ*_max_ (log *ε*) = 249 (3.99) and 202 (3.74) nm; IR *ν*_max_ = 3392, 2973, 2951, 2867, 1740, 1736, 1660, 1620, 1460, 1433, 1378, 1337 cm^–1^; for ^1^H NMR (400 MHz) and ^13^C NMR (100 MHz) data see Tables S1;† HRESIMS [M + Na]^+^ *m*/*z* 451.2410 (calcd for C_26_H_36_O_5_Na, 451.2460).

#### Compound **2**

Optically active white gum, [*α*]20D +44.2 (*c* = 1.5, MeOH); ECD (CH_3_OH) *λ* (Δ*ε*) 209 (+8.83), 246 (–5.78), 259 (+0.94) nm, 309 (–0.56) nm, 345 (+2.11); UV (MeOH) *λ*_max_ (log *ε*) = 253 (3.12) and 209 (3.31) nm; IR *ν*_max_ = 3396, 3263, 2957, 2881, 1741, 1725, 1711, 1453, 1433, 1388, 1368, 1345 cm^–1^; for ^1^H NMR (400 MHz) and ^13^C NMR (100 MHz) data see Tables S1;† HRESIMS [M + Na]^+^ *m*/*z* 499.2637 (calcd for C_27_H_40_O_7_Na, 499.2672).

#### Compound **3**

Colorless crystals, [*α*]20D –84.5 (*c* = 2, MeOH); UV (MeOH) *λ*_max_ (log *ε*) = 231 (3.28) and 202 (3.81) nm; IR *ν*_max_ = 3528, 2990, 2944, 1776, 1754, 1728, 1618, 1446, 1387 cm^–1^; for ^1^H NMR (400 MHz) and ^13^C NMR (100 MHz) data see Tables S1;† HRESIMS [M + Na]^+^ *m*/*z* 481.2199 (calcd for C_26_H_34_O_7_Na, 481.2202).

#### X-ray crystal data for **1**

The crystal structure and absolute configuration of **1** were determined using data collected at *T* = 296 K with Cu Kα radiation (*λ* = 1.54178 Å) on a Bruker APEX-II CCD diffractometer. The structure was solved using the direct method with SHELXS-97 and refined with full-matrix least-squares calculations on *F* ^2^ by using SHELXS-97.^26^ All non-hydrogen atoms were refined anisotropically. The hydrogen atom positions were geometrically idealized and allowed to ride on their parent atoms. The absolute configuration was determined by refinement of the Flack parameter based on resonant scattering of the light atoms and computation of the Hooft parameter, in all cases yielding a probability of 1.000 that the reported configuration is correct.^27,28^

#### Animal experiments

The generation and characterization of the 3xTg-AD mouse model, as well as the timing of disease progression, were previously described.^29^ All of the animal experiments were performed according to the ‘Policies on the Use of Animals and Humans in Neuroscience Research’. The animals were fed in a room under standard housing conditions (room temperature 24 to 27 °C, humidity 60–65% and 12 h light–dark cycle) with free access to food and water. The mice were aged 4 to 5 months and weighed 20 to 25 g. The mice were divided randomly into four groups (*n* = 10 in each). The mice were anesthetized with 6% chloral hydrate (6 mL kg^–1^, i.p.) and different solutions (compound **1** 2 μg μL^–1^ × 5 μL, 0.2 μg μL^–1^ × 5 μL, LY2811376 2 μg μL^–1^ × 5 μL, DMSO 5 μL as control) were infused very slowly into the cerebral ventricles of the mice with the following coordinates: 0.2 mm anterior to posterior (AP) bregma, 0.9 mm midline to lateral (ML), and 2.3 mm dorsal to the ventral (DV) dura. After 48 hours, a water maze was used to train and test the ability of spatial learning and memory of mice. After this procedure, which was carried out for 6 days, the mice were sacrificed and other tests were performed.

#### Morris water maze assay

Spatial learning and memory were measured using the Morris water maze, according to the classic Morris protocol.^30^ The water maze used for the mice was a plastic, circular pool 2.1 m in diameter and 0.6 m in height that was filled with water (temperature at 25 °C ± 1 °C). A circular platform (13 cm in diameter) was placed at a specific location away from the edge of the pool. The top of the platform was submerged 1.5 cm below the water surface. Water was made cloudy by adding milk powder. Distinctive visual cues were set on the wall. For spatial learning and training, animals were subjected to four trials a day in the afternoon. The training procedure lasted a further 4 days and a total of 20 trials were performed. For each trial, the animal was placed at a different starting position, spaced equally around the perimeter of the pool. Animals were given 60 s to find the platform. If an animal could not find the platform, it was guided to the platform and allowed to stay there for 30 s. The time that each animal took to reach the platform was recorded as the escape latency. A probe trial of 60 s was carried out one day after the end of training to test memory retention. For the probe trial test, animals were placed in the pool with the platform removed and the time they spent in the target quadrant and the number of times that they crossed the platform’s location were recorded.

#### Cell culture and compound treatment

The N2a-APP cells were cultured in Dulbecco's modified Eagle's medium (DMEM)/Opti-MEM (1 : 1, v/v), supplemented with 10% fetal bovine serum (FBS, Gibco BRL, Gaithersburg, MD, USA) and 200 μg mL^–1^ G418, and grown at 37 °C in a humid atmosphere with 5% CO_2_/95% air. Cells were seeded in 6-well culture plates (1 × 10^6^ cells per well).^21^ After adherence and confluence were reached, the medium in each well was replaced with 1000 μL of the appropriate fresh medium, containing the test compound in DMSO or the vehicle alone in control wells. After 24 h of treatment, further analysis was continued.

#### MTT assay

The neuroprotective activities of these isolated compounds were measured according to a published procedure.^31^ HEK293 cells were cultured in Dulbecco's modified Eagle's medium (DMEM) supplemented with 10% fetal bovine serum (FBS, Gibco BRL, Gaithersburg, MD, USA) and grown at 37 °C in a humid atmosphere containing 5% CO_2_. Cells were seeded in 96-well culture plates (1 × 10^4^ cells per well). After 24 h of incubation, the cells were treated with compounds at different concentrations. After 24 h of treatment, 10 μL of MTT (5 mg mL^–1^) was added. For the MTT assays, the supernatant was discarded and DMSO (100 μL per well) was added. After 30 min, the optical density at 450 nm was measured using a microplate reader.

#### Golgi staining

The Golgi staining protocol was performed as previously described.^32^ Briefly, the mice were anesthetized after the behavioural tests and their aortae were perfused with approximately 200 mL of normal saline containing 0.5% sodium nitrite, followed by 200 mL of 4% formaldehyde solution and 200 mL Golgi fixative (5% chloral hydrate, 4% formaldehyde and 5% potassium dichromate) for 4 h in the dark. The brains were incubated in the same Golgi fixative for 3 days and transferred to a silver solution containing 1% silver nitrate for 3 days in the dark. Coronal brain sections of hippocampus tissue were cut into 35 μm sections using a vibrating microtome (Leica, VT1000S and Germany).

#### Nissl staining

For the Nissl staining,^33^ 35 μm-thick sections mounted on gelatin-coated slides were incubated in toluidine blue (pH 4.1) for 2 min, dehydrated through a battery of alcohols of increasing concentration (75%, 80%, 85%, 90%), and cover slipped in Eukitt for 10 min twice.

#### Western blotting

Hippocampi were rapidly removed from the brains and homogenized in a buffer containing 10 mM Tris–Cl (pH 7.6), 1 mM Na_3_VO_4_, 50 mM NaF, 1 mM benzamidine, 1 mM EDTA, and 1 mM phenylmethylsulfonyl fluoride (PMSF).^34^ The homogenates were mixed with one-third of sample buffer (200 mM Tris–HCl, 8% sodium dodecyl sulfate and 40% glycerol), boiled for 10 min, and centrifuged at 14 000*g* for 10 min. For cell samples, cells were washed with PBS and lysed with 1× RIPA buffer. The protein concentrations of the supernatants were measured using the BCA method. The same amount of protein was separated using SDS-polyacrylamide gel electrophoresis (10%) and transferred to a nitrocellulose membrane. The membranes were blocked in 3% non-fat milk for 1 h and incubated with the following primary antibodies at 4 °C overnight: polyclonal anti-human sAPPβ antibodies from IBL (dilution 1 : 1000, Aramachi, Gunma, JAPAN); polyclonal anti-APP antibodies from Cell Signaling Technology (dilution 1 : 1000, Beverly, MA, USA); polyclonal anti-PSD95 antibodies obtained from Abcam, (dilution 1 : 1000, Cambridge, MA, USA); polyclonal anti-PSD93 antibodies obtained from Abcam (dilution 1 : 1000, Cambridge, MA, USA); monoclonal anti-β-actin antibodies obtained from Abcam (dilution 1 : 1500 Cambridge, MA, USA); polyclonal synapsin-1 antibodies obtained from Millipore (dilution 1 : 1000, Temecula, CA, USA); and monoclonal anti-synaptophysin antibodies obtained from Sigma (dilution 1 : 1000, MO, USA). The membranes were incubated with secondary antibody conjugated to IRDye (800 CW) for 2 h and visualized using the Odyssey Infrared Imaging System (LI-Cor Biosciences, Lincoln, NE, USA).

#### BACE1 enzymatic assay

The enzyme inhibition assay was carried out using β-Secretase Activity Assay Kit from Abnova according to the manufacturer instructions and using a multi-well fluorescence plate reader that allowed absorption at 335–355 nm and recorded emission at 495–510 nm.^35^ The assay procedure was conducted as follows: the lyophilized Active β-secretase was reconstituted by adding 10 μL of ddH_2_O to make a working solution. 50 μL lysate was added to each well in a 96-well plate. 50 μL of 2× reaction buffer and 2 μL of β-secretase substrate were added. The reaction mixtures were incubated in the dark at 37 °C for 1 hour. Fluorescence was monitored at 334–355 nm (excitation wavelength) and 490–510 nm (emission wavelength).

#### ELISA

Secreted Aβ_42_ was measured in conditioned medium and tissue using commercial human Aβ_42_ ELISA kits (Elabscience, Wuhan, China) according to the manufacturer's instructions.

#### BACE-1 docking protocol

Docking calculations were performed with Glide XP, applying post dock strain penalties.^36,37^ Nitrogen inversion or ring conformations were sampled and non-planar conformations of amides were penalized and Epik states penalties were added and as output options the number of poses to save was increased to 10 and expanded sampling option was adopted. The binding mode in BACE-1 is strictly dependent on the protonation state. For further analysis, we selected the structures that give the best score, and the mono-protonated (32i) 1YM2 performed better than the rest.

#### Statistical analysis data

Data are presented as mean ± standard deviation. Data were analyzed using one-way analysis of variance with a *post hoc* test (multiple comparison test), which determined the significant differences among groups. When *P* < 0.05, the difference is considered significant.
