# Supplementary material for: Asperterpenes A and B, two unprecedented meroterpenoids from Aspergillus terreus with BACE1 inhibitory activities
Source: Chem Sci. 2016 Jun 27;7(10):6563–72. doi: 10.1039/c6sc02464e (PMC5131395; doi:10.1039/c6sc02464e)
Supplement: Supplementary file 1 [file SC-007-C6SC02464E-s001.pdf]

Electronic Supplementary Material (ESI) for Chemical Science.
This journal is © The Royal Society of Chemistry 2016

Asperterpenes A and B, Two Unprecedented Meroterpenoids with BACE1 Inhibitory Activities from *Aspergillus terreus*

Changxing Qi,† Jian Bao,† Jianping Wang,† Hucheng Zhu, Yongbo Xue, Xiaochuan Wang, Hua Li, Weiguang Sun, Weixi Gao, Yongji Lai, Jian-Guo Chen, and Yonghui Zhang*

[a] C. Qi, J. Wang, H. Zhu, Y. Xue, H. Li, W. Sun, Y. Lai, W. Gao and Y. Zhang

Hubei Key Laboratory of Natural Medicinal Chemistry and Resource Evaluation, School of Pharmacy, Tongji Medical College, Huazhong University of Science and Technology, Wuhan 430030, P. R. China; E-mail: zhangyh@mails.tjmu.edu.cn (Y.Z.).

[b] J. Bao, X. Wang, J. G. Chen

School of Basic Medicine, Tongji Medical College, Huazhong University of Science and Technology, Wuhan 430030, China

† These authors contributed equally to this work.

Supporting information for this article is available on the WWW under http://

CONTENTS

Table S1.^{1}H and ^{13}C NMR Data of compounds **1-3** in $CDCl_3$......................................3

Table S2. Predicted binding free energies of **1** and **2** in AD involved targets..............4

Table S3. BACE1 Inhibitory activities of compounds **1**–**3** in vitro..............................4

Figure. S1 Representative micrographs of Nissl staining assay of **1**. 5

Figure. S2 Speed to find the hidden platform in morris mize of **1**................................5

Figure. S3 MTT assay of compounds **1**-**3**...6

Figure. S4 Key 2D HMBC correlations of compounds **1**-**3**...6

Figure. S5 Key 2D NOESY correlations of compounds **1**-**3**. 7

Figure. S6 The structure of LY2811376. 7

Crystal data for compound **1**..8

Crystal data for compound **3** 8

HRESIMS, NMR, UV, IR and CD spectra of **1**...10

HRESIMS, NMR, UV, IR and CD spectra of **2**...20

HRESIMS, NMR, UV, IR and CD spectra of **3**...30

^{1}H and ^{13}C NMR spectra of **4**..40

Table S1. NMR data of compounds **1–3** (in $CDCl_3$, 400 MHz for ^1H and 100 MHz for ^{13}C)

no.	**1**			**2**			**3**		
	δ_H (*J* in Hz)	δ_C	DEPT	δ_H (*J* in Hz)	δ_C	DEPT	δ_H (*J* in Hz)	δ_C	DEPT
1a	1.98 m	30.6	CH_2	1.97 m	31.9	CH_2	2.14 m	40.3	CH_2
1b	1.35 m			1.48 m			1.55 m		
2a	2.64 ddd (15.8, 12.0, 6.1)	33.0		2.20 m	30.1		2.78 m	33.4	
2b	2.26 ddd (15.8, 9.8, 2.6)		CH_2			CH_2	2.24 m		CH_2
3		218.8	C		174.6	C		213.6	C
4		47.0	C		75.0	C		46.8	C
5	2.36 dd (13.2, 2.3)	44.0	CH	1.44 m	47.0	CH	2.67 s	64.1	CH
6a	1.54 m	18.8	CH_2	1.73 m	21.4	CH_2		209.1	CH_2
6b	1.34 m								
7a	1.98 m	32.2		1.90 m	31.7		4.07 d (13.2)	52.1	CH_2
7b	1.37 m		CH_2	1.24 m		CH_2	2.79 d (13.2)		
8		46.3	C		46.3	C		44.5	C
9	1.30 m	49.4	CH	2.18 m	48.7	CH	1.85 dd (13.7, 3.7)	50.7	CH
10		36.6	C		41.4	C		42.3	C
11a	2.18 t (13.7)	39.8	CH_2	2.24 m	36.9		2.50 dd (16.1, 3.5)	28.2	
11b	1.82 dd (13.7, 2.8)			1.88 m		CH_2	2.71 m		CH_2
12		49.5	C		49.3	C		142.5	C
13		163.9	C		162.2	C		57.8	C
14		131.3	C		131.9	C		72.6	C
15		198.2	C		197.4	C		210.1	C
16		67.4	C		68.7	C		74.7	C
17		210.2	C		210.7	C		210.2	C
18	1.05 s	29.3	CH_3	1.35 s	32.0	CH_3	1.15 s	24.0	CH_3
19	0.99 s	19.2	CH_3	1.35 s	35.0	CH_3	1.52 s	21.8	CH_3
20	1.34 s	25.2	CH_3	1.28 s	20.0	CH_3	1.41 s	19.8	CH_3
21	0.74 s	22.4	CH_3	1.14 s	24.7	CH_3	1.22 s	16.2	CH_3
22	1.28 s	18.6	CH_3	1.29 s	20.9	CH_3	5.12 s; 4.95 s	117.2	CH_2
23	2.02 s	17.7	CH_3	2.02 s	17.6	CH_3	1.56 s	23.2	CH_3
24	1.18 s	14.2	CH_3	1.17 s	12.6	CH_3	1.43 s	25.3	CH_3
25		166.8	C		166.9	C		168.0	C
26	3.82 s	52.5	OCH_3	3.82 s	52.4	OCH_3	3.69 s	52.9	OCH_3
27				3.66 s	51.7	OCH_3			

Table S2. Predicted binding free energies of compounds and target (MF docking scores) [a]

PDB-ID	Protein name	Compounds	
		1	2
4Y6K	PSH	-140.2	-176.5
4QO6	GlpG	-77.0	-73.8
4J36	KMO	-110.4	-51.4
2MXU	Amyloid Fibril	-138.5	-109.2
1GS9	APOE4	-57.9	-68.5
4NF5	NMDA receptor	-81.9	-85.1
4EY6	Acetylcholinesterase	-100.3	-75.2
4XXS	BACE1	-193.9	-178.8

[a] Docking score/interaction potential of compounds with targets (kcal/mol).

Table S3. Inhibitory activities of compounds **1**–**3**

Compound	Inhibitory activities against BACE1
	IC_{50} (nM)
**1**	78.8
**2**	59.1
**3**	n.i.[a]
LY2811376	260.2

[a] n.i. is no inhibition detected in the experiments (IC_{50}□40 μM).

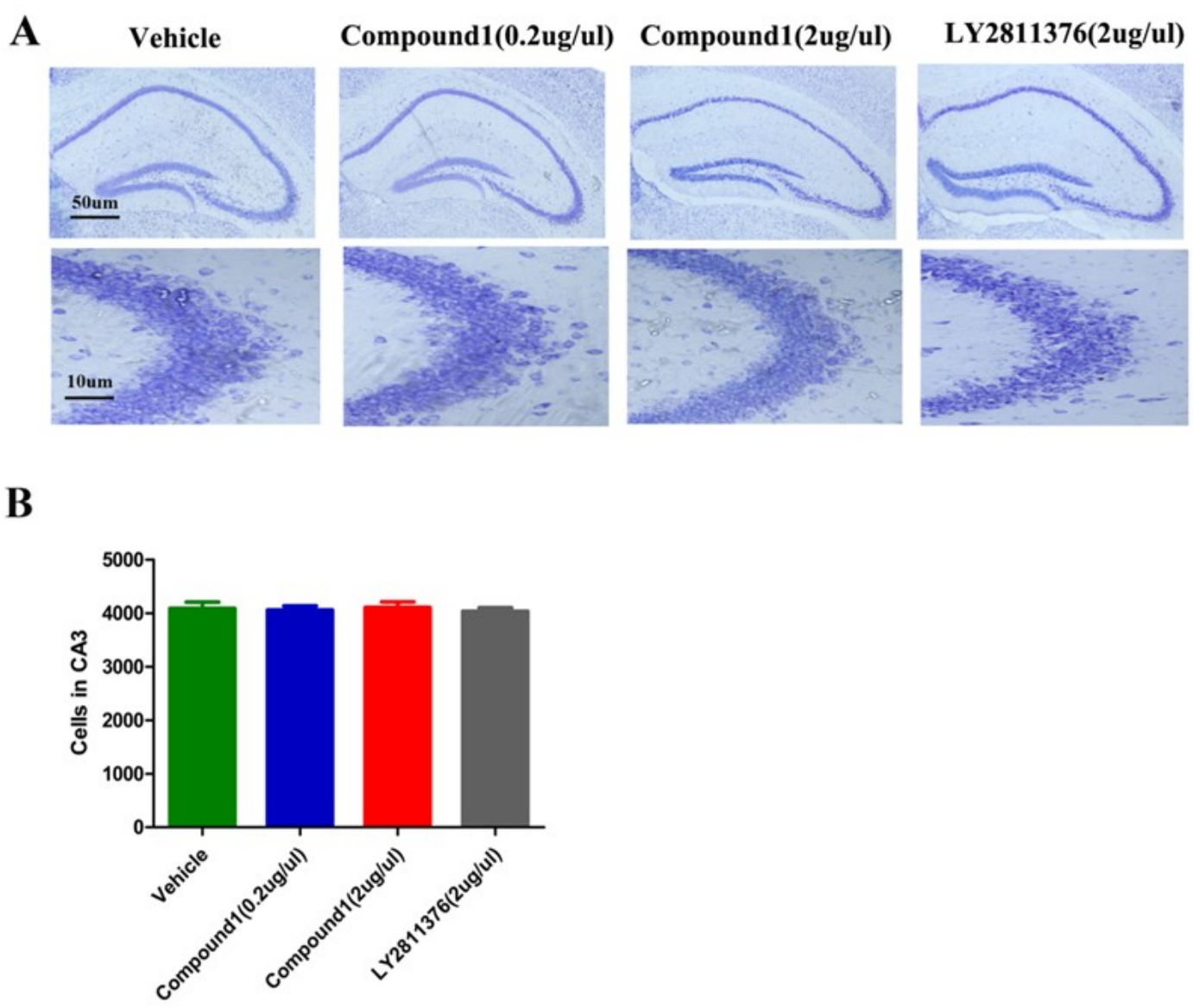

Figure S1. Nissl staining of the cells numbers in hippocampus from compound **1** (2 μg/μL×5μL，0.2 μg/μL×5μL), LY2811376 (2 μg/μL×5μL) and vehicle groups. A) Nissl staining (upper row, cells in hippocampus; lower row, cells in CA3. B) the quantification of cells in CA 3. The data were expressed as mean ± SD, (n= 3).

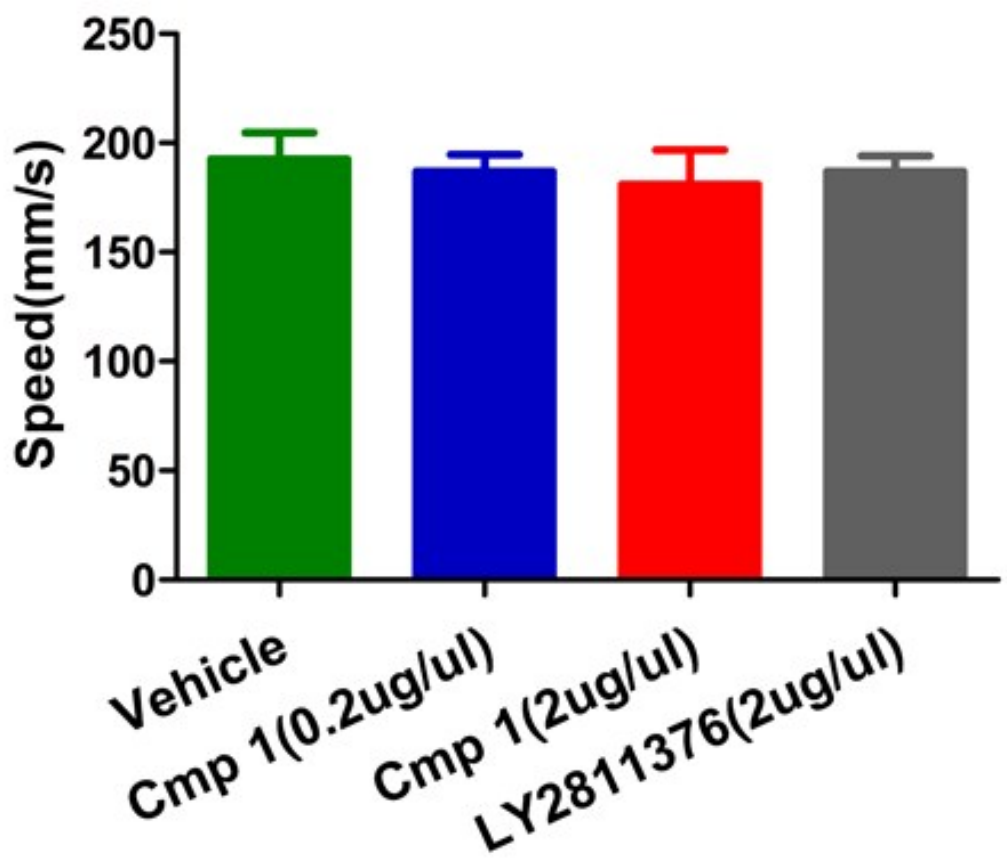

Figure S2. The effects of 1 on learning and memory in 3xTg mice. Compound **1** (2 μg/μL×5μL, 0.2 μg/μL ×5μL), LY2811376 (2 μg/μL×5μL) or vehicle was infused into the cerebroventricles of 3xTg mice 48 hours before starting the task. The speed to find the hidden platform was recorded daily. Data are presented as means ± sd (n = 7).

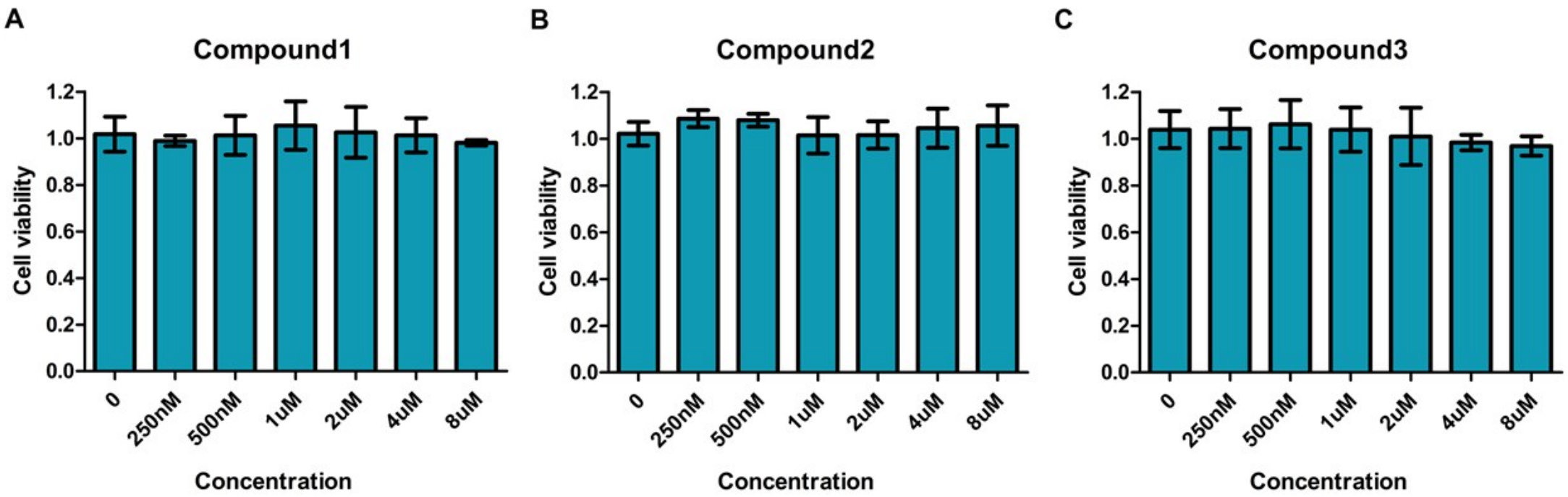

Figure S3. Cell viability was assessed through MTT assay. HEK293 cells were treated with 0, 0.25, 0.5, 1, 2, 4 and 8 μM. Data represent mean±SD. Student t-Test compared to cell treated with vehicle alone.

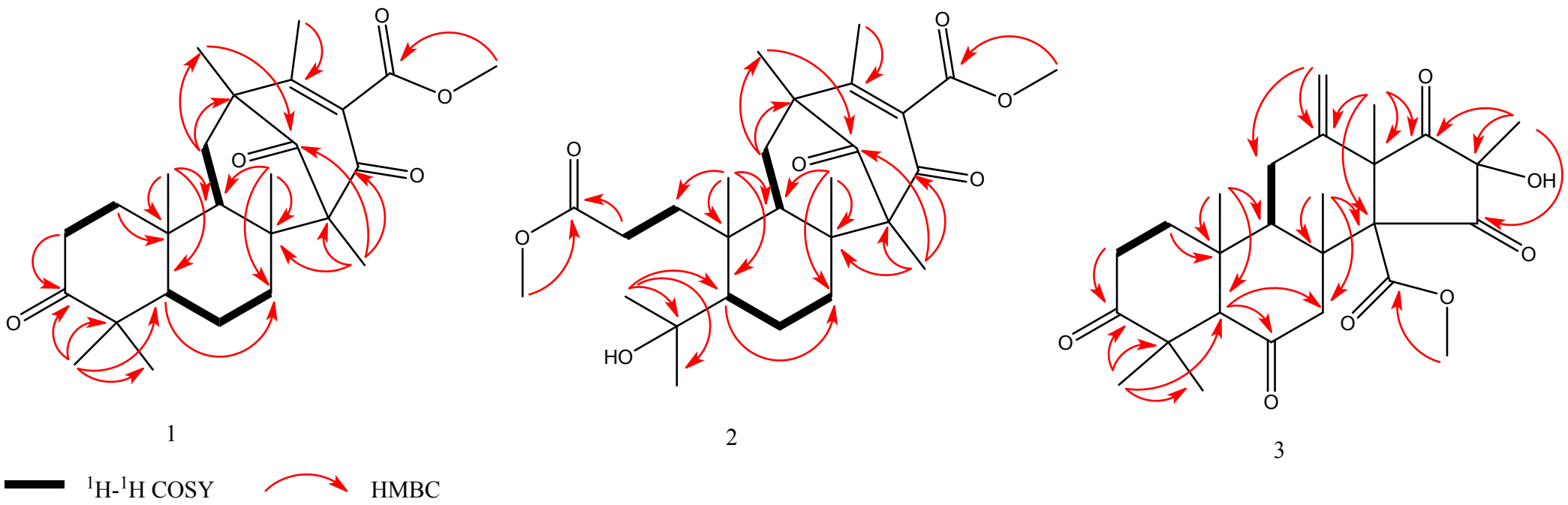

Figure S4. Key HMBC correlations of **1-3**.

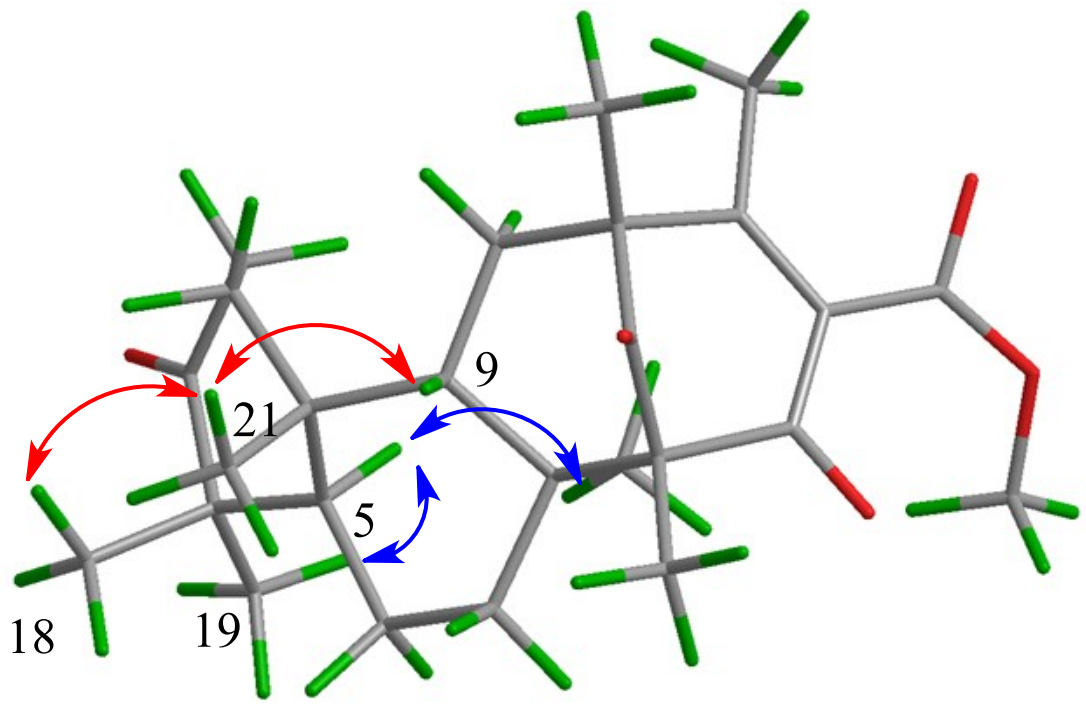

1

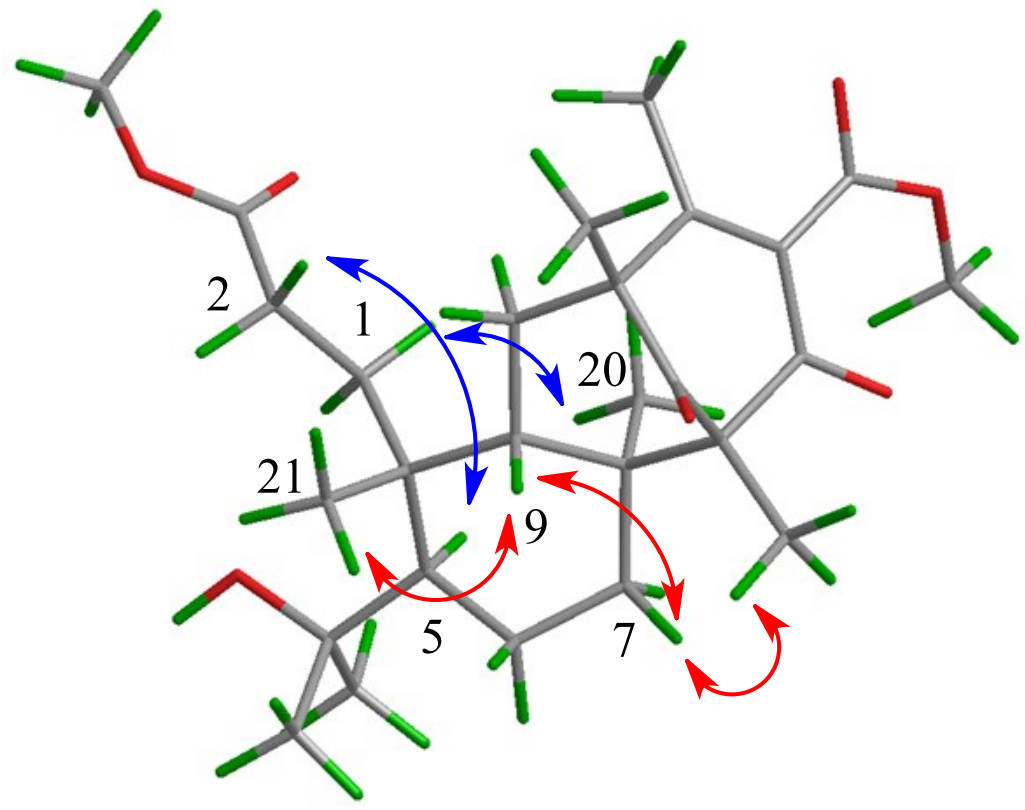

2

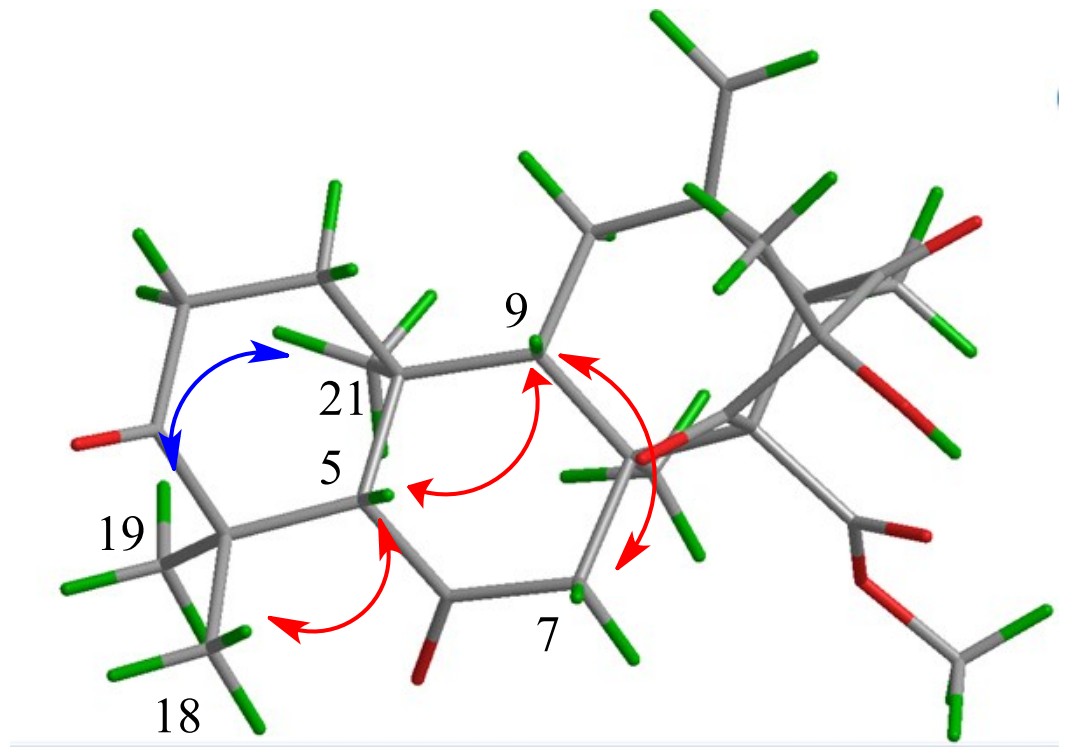

3

Figure S5. Key NOESY correlations of **1-3**.

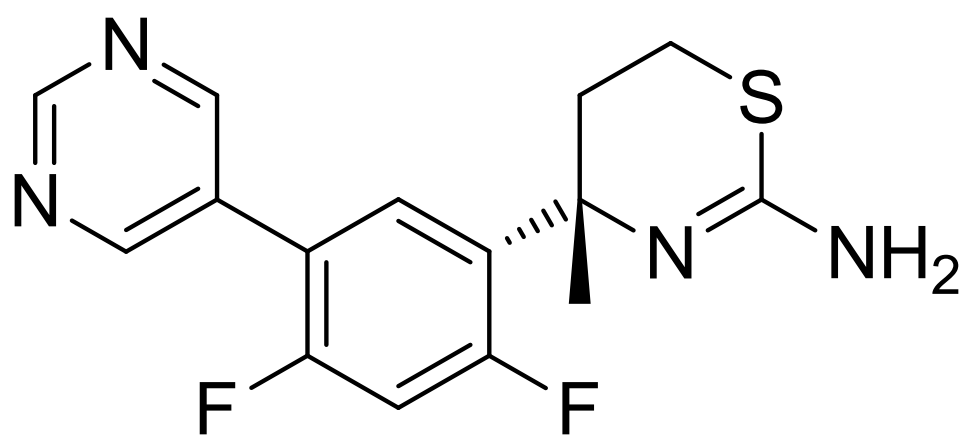

Figure S6. The structure of LY2811376.

Crystal data for compound 1: $C_{26}H_{36}O_5$, M = 428.55, orthorhombic, a = 7.9305(2) Å, b = 11.6311(3) Å, c = 25.2575(5) Å, α = 90.00°, β = 90.00°, γ = 90.00°, V = 2329.76(10) Å^3, T = 296(2) K, space group P212121, Z = 4, μ(CuKα) = 0.666 mm^{-1}, 9987 reflections measured, 3701 independent reflections (R_{int} = 0.0304). The final R_1 values were 0.0354 ($I > 2\sigma(I)$). The final $wR(F^2)$ values were 0.1015 ($I > 2\sigma(I)$). The final R_1 values were 0.0358 (all data). The final $wR(F^2)$ values were 0.1021 (all data). The goodness of fit on F^2 was 1.080. Flack parameter = 0.0(2). The Hooft parameter is -0.02(7) for 1531 Bijvoet pairs. (CCDC 1416500)

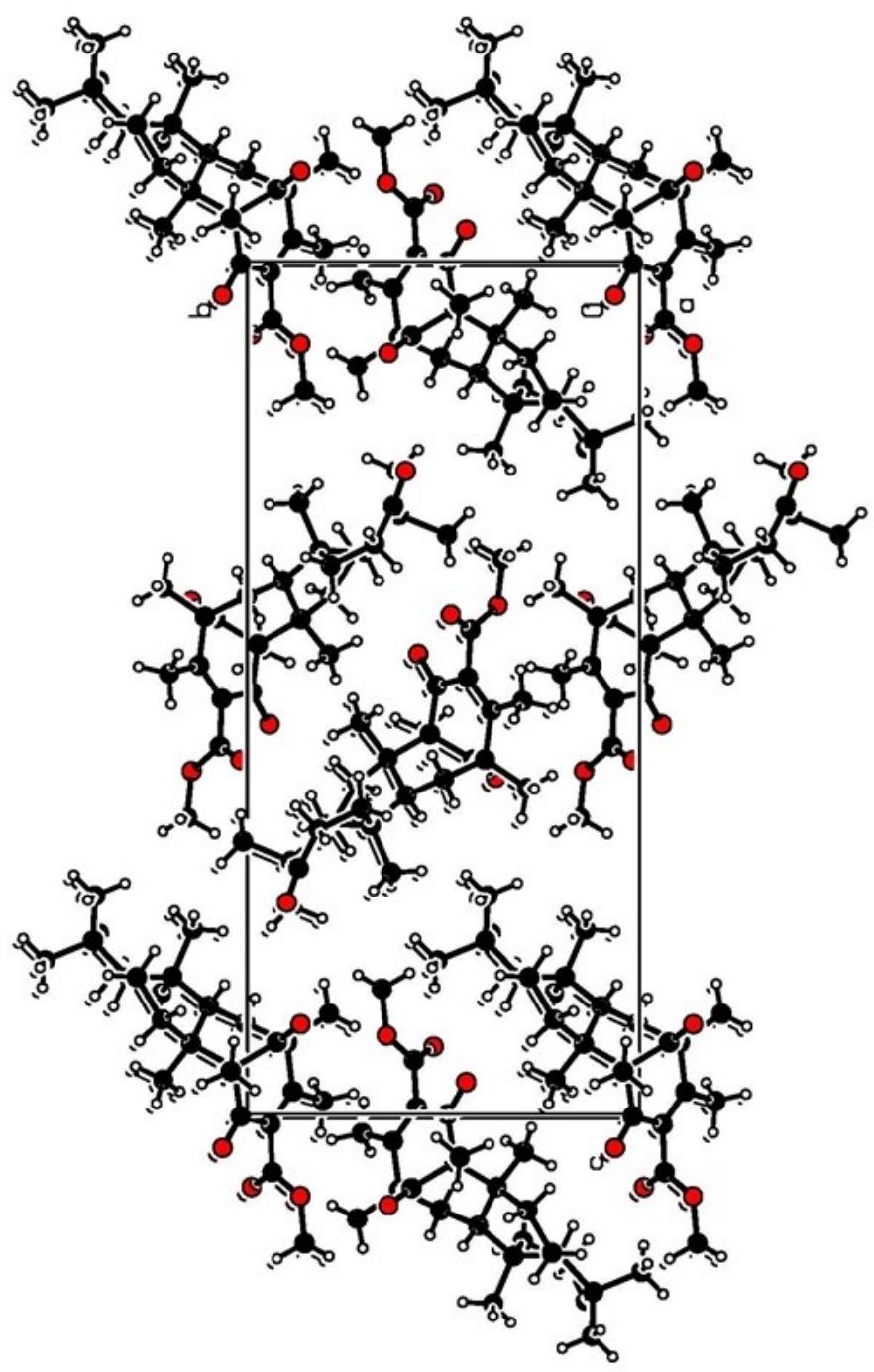

View of the hydrogen-bonded motif of **1** (hydrogen-bonds are shown as dashed lines)

Crystal data for compound 3: $C_{26}H_{36}O_7$, M = 474.55, orthorhombic, a =15.9092(4) Å, b = 18.9397(5) Å, c = 8.0829(2) Å, α = 90.00°, β = 90.00°, γ = 90.00°, V = 2435.50(11) Å^3, T = 298(2) K, space group P2(1)2(1)2, Z = 4, μ(CuKα) = 0.770 mm^{-1}, 13869 reflections measured, 3931 independent reflections ([R(int) = 0.0479]). The final R_1 values were 0.0407 ($I > 2\sigma(I)$). The final $wR(F^2)$ values were 0.1136 ($I > 2\sigma(I)$). The final R_1 values were 0.0411 (all data). The final $wR(F^2)$ values were 0.1140 (all data). The goodness of fit on F^2 was 1.046. Flack parameter = 0.14(18). The Hooft parameter is 0.0119(8) for 1939 Bijvoet pairs. （CCDC 1416501）

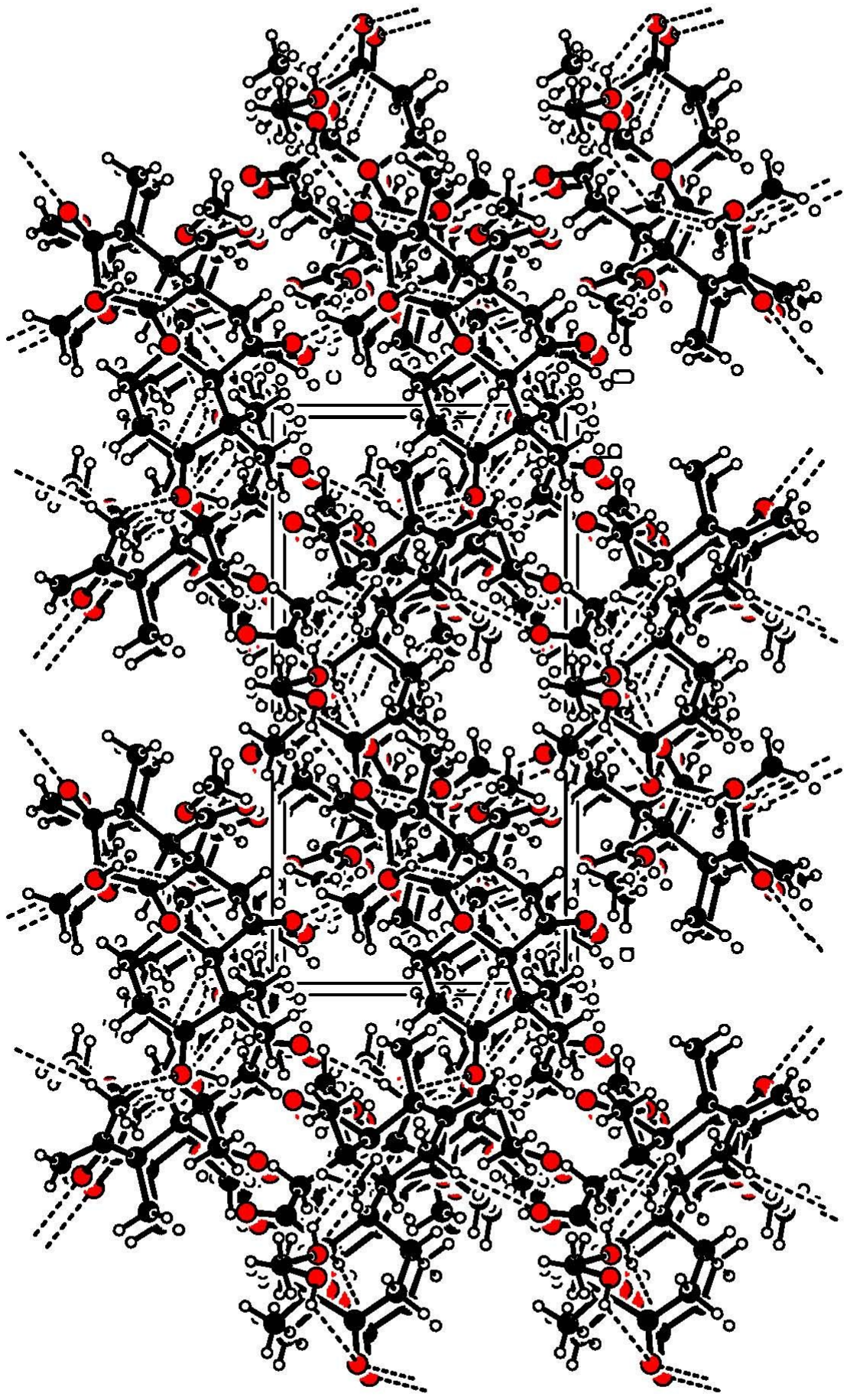

View of the hydrogen-bonded motif of **3** (hydrogen-bonds are shown as dashed lines)

Compound 1: Colorless crystals, $[\alpha]^{20}_{D}$ +47.7 (*c* = 0.9, MeOH); UV (MeOH) λ_{max} (log ε) = 249 (3.99) and 202 (3.74) nm; for ^{1}H NMR (400 MHz) and ^{13}C NMR (100 MHz) data see Tables S1; HRESIMS [M + Na]$^{+}$ *m/z* 451.2410 (calcd for $C_{26}H_{36}O_{5}Na$, 451.2460).

Compound 2: Optically active white gum, $[\alpha]^{20}_{D}$ +44.2 (*c* = 1.5, MeOH); UV (MeOH) λ_{max} (log ε) = 253 (3.12) and 209 (3.31) nm; for ^{1}H NMR (400 MHz) and ^{13}C NMR (100 MHz) data see Tables S1; HRESIMS [M + Na]$^{+}$ *m/z* 499.2637 (calcd for $C_{26}H_{36}O_{5}Na$, 499.2672).

Compound 3: Colorless crystals, $[\alpha]^{20}_{D}$ -84.5 (*c* = 2, MeOH); UV (MeOH) λ_{max} (log ε) = 231 (3.28) and 202 (3.81) nm; for ^{1}H NMR (400 MHz) and ^{13}C NMR (100 MHz) data see Tables S1; HRESIMS [M + Na]$^{+}$ *m/z* 481.2199 (calcd for $C_{26}H_{34}O_{7}Na$, 881.2202).

HRESIMS of compound **1**

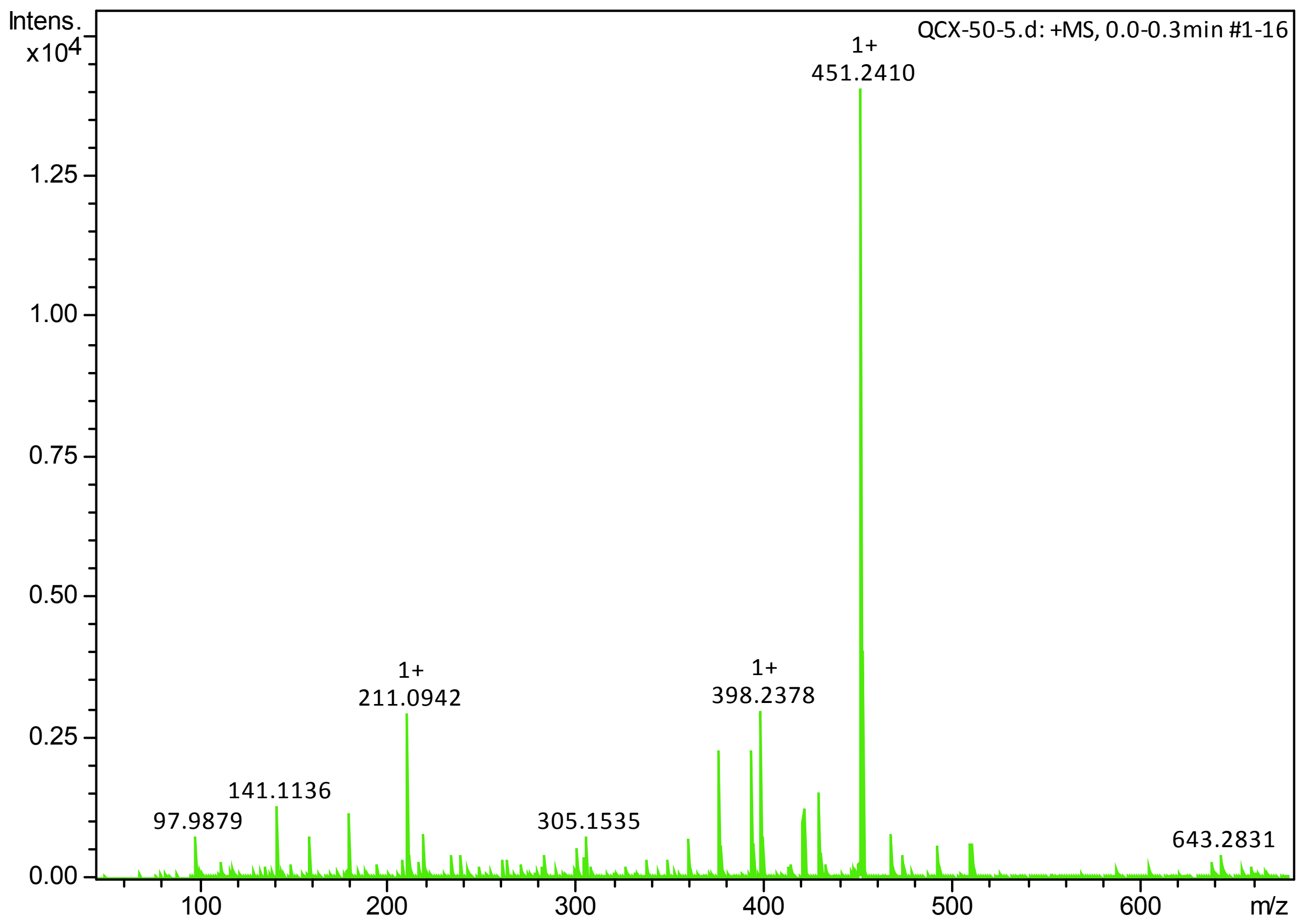

^{1}H NMR of compound **1** (in $CHCl_3$)

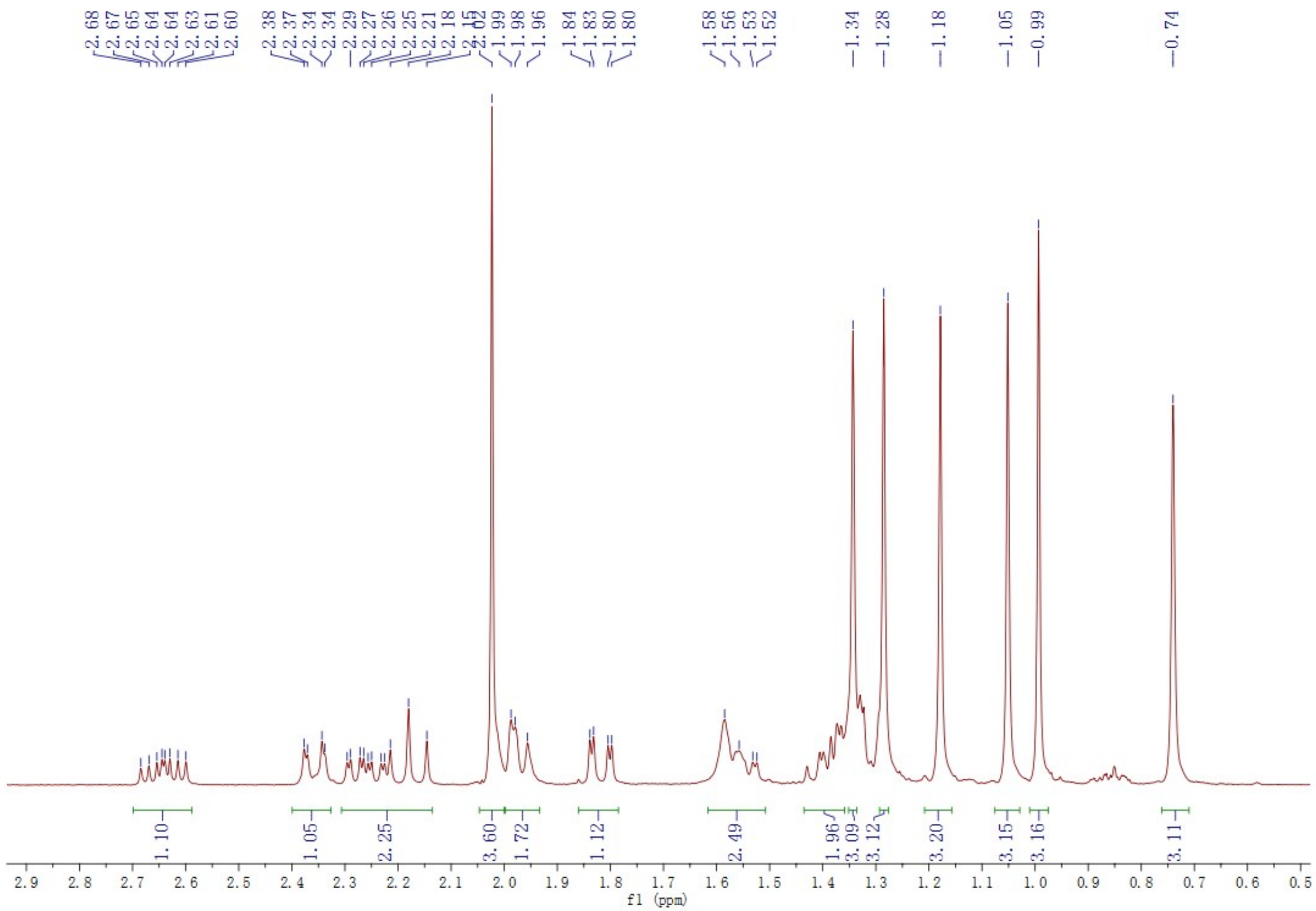

^{13}C NMR of compound **1** (in $CHCl_3$)

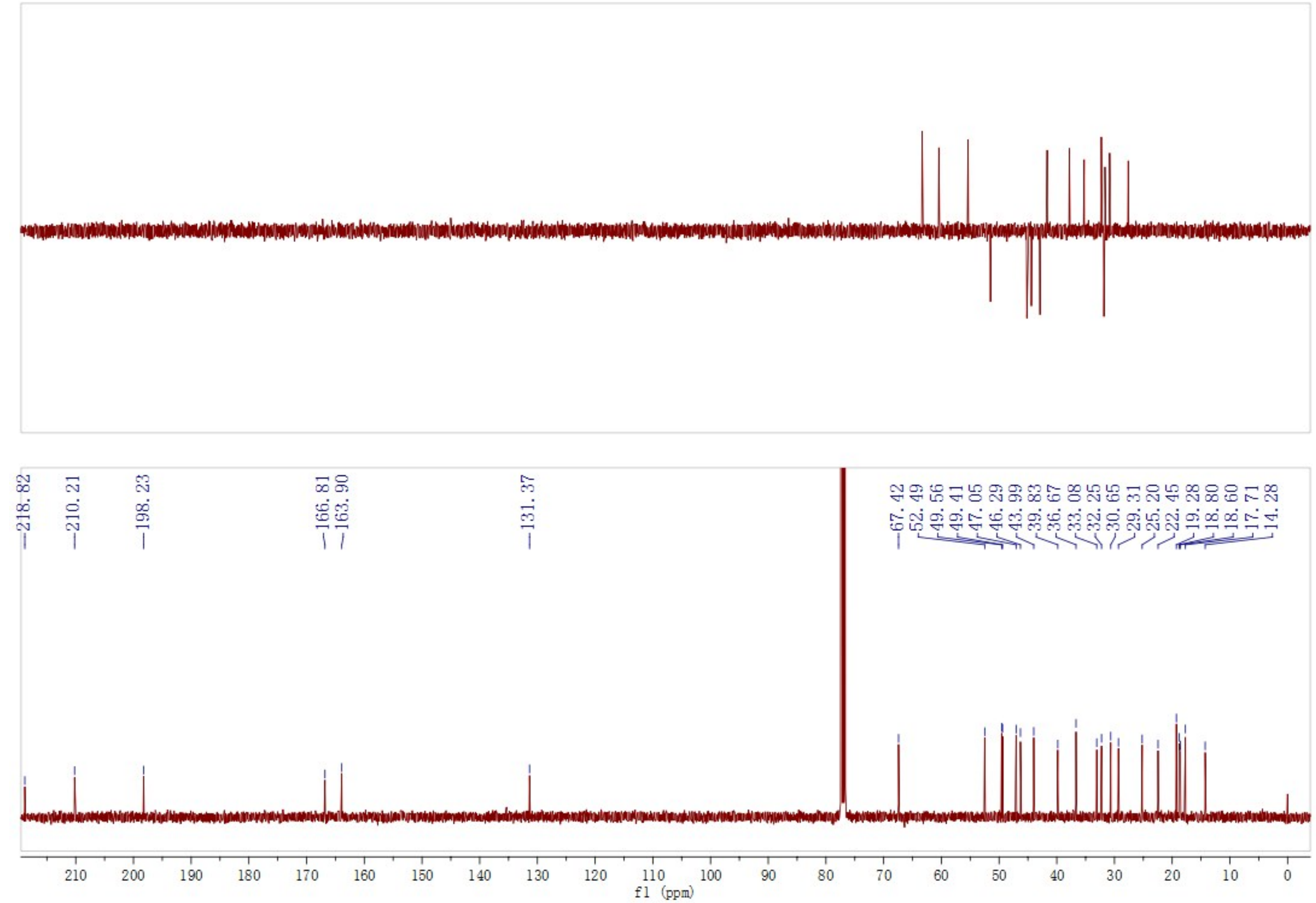

HSQC of compound **1** (in $CHCl_3$)

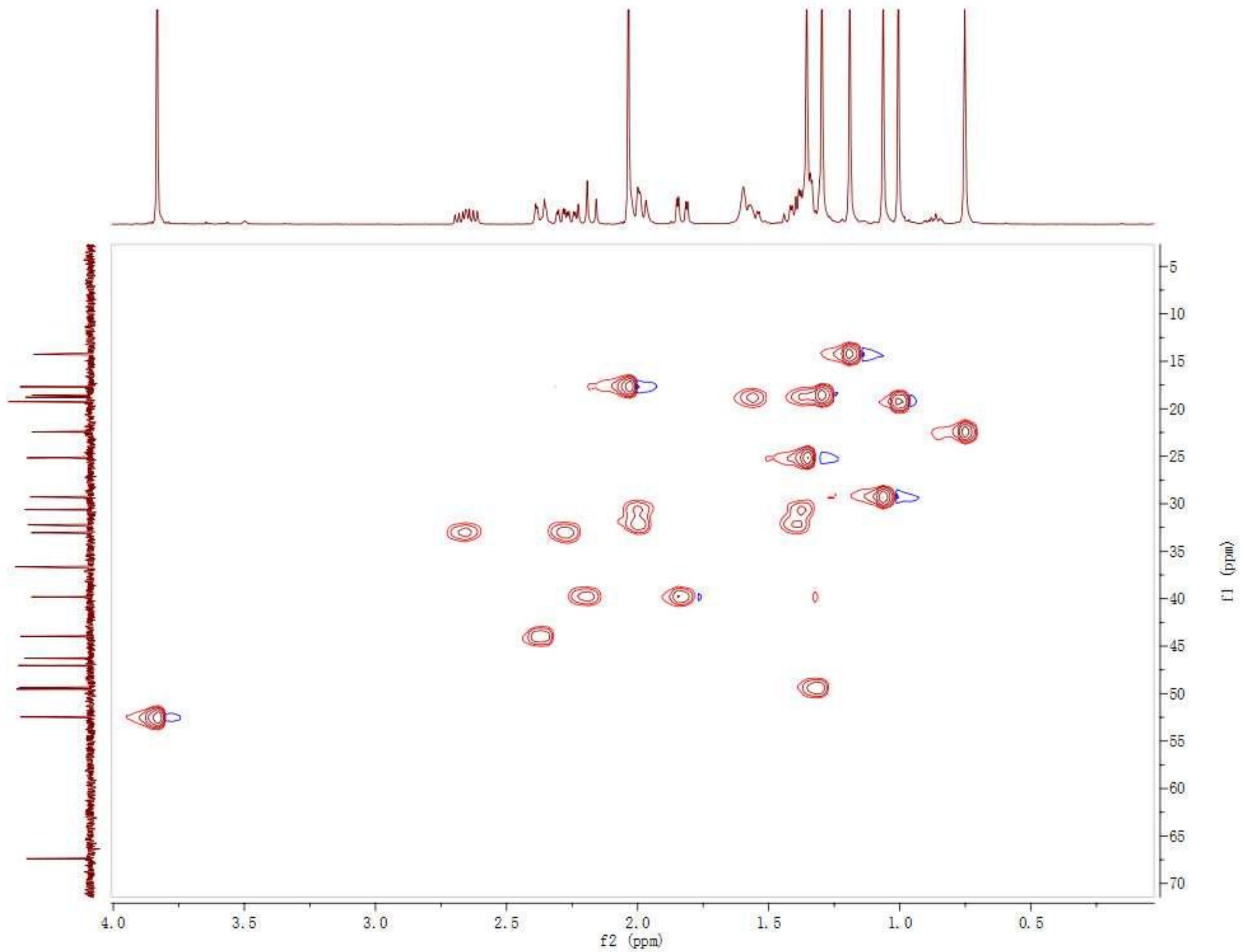

HMBC of compound **1** (in $CHCl_3$)

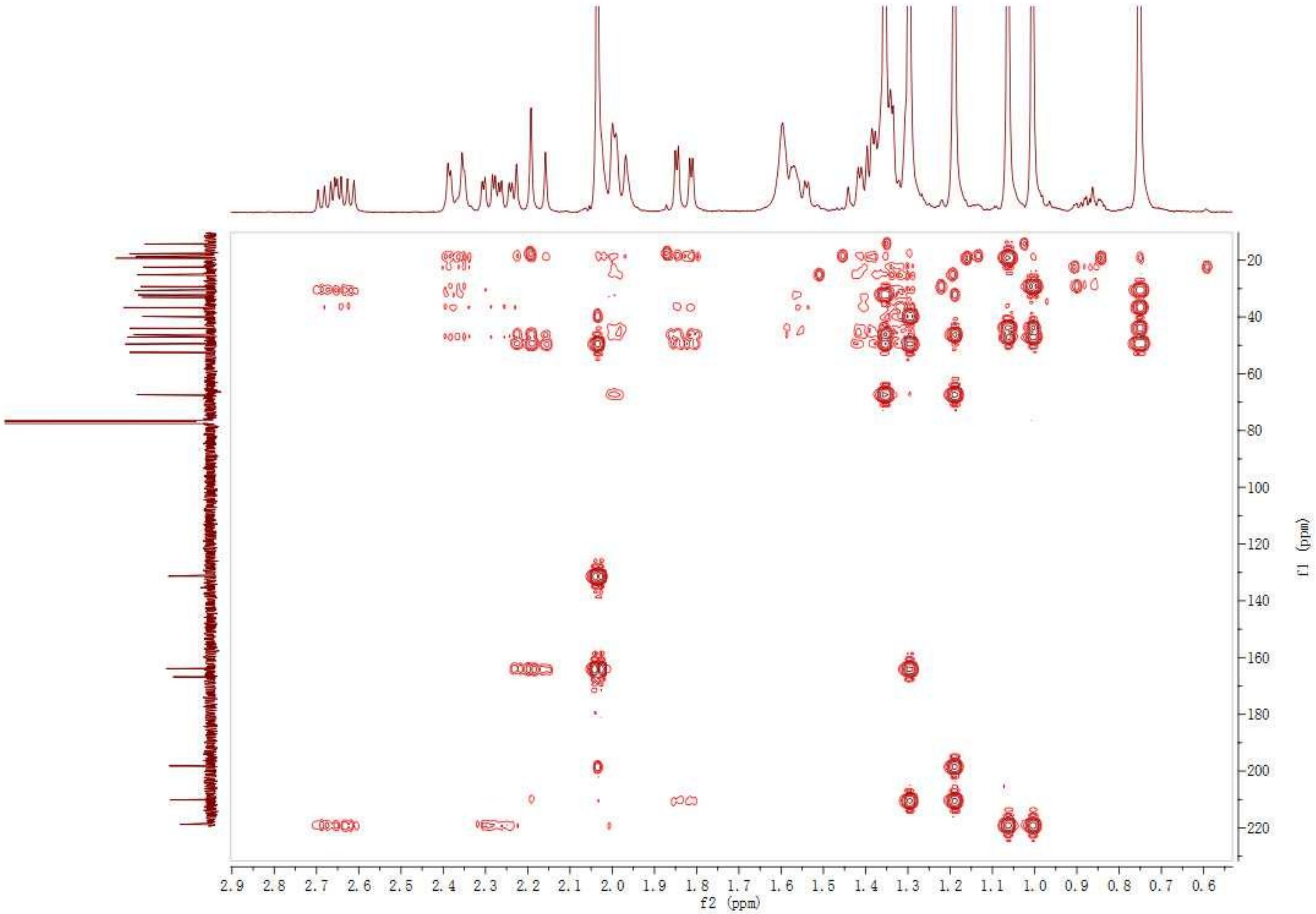

^{1}H–^{1}H COSY of compound **1** (in $CHCl_3$)

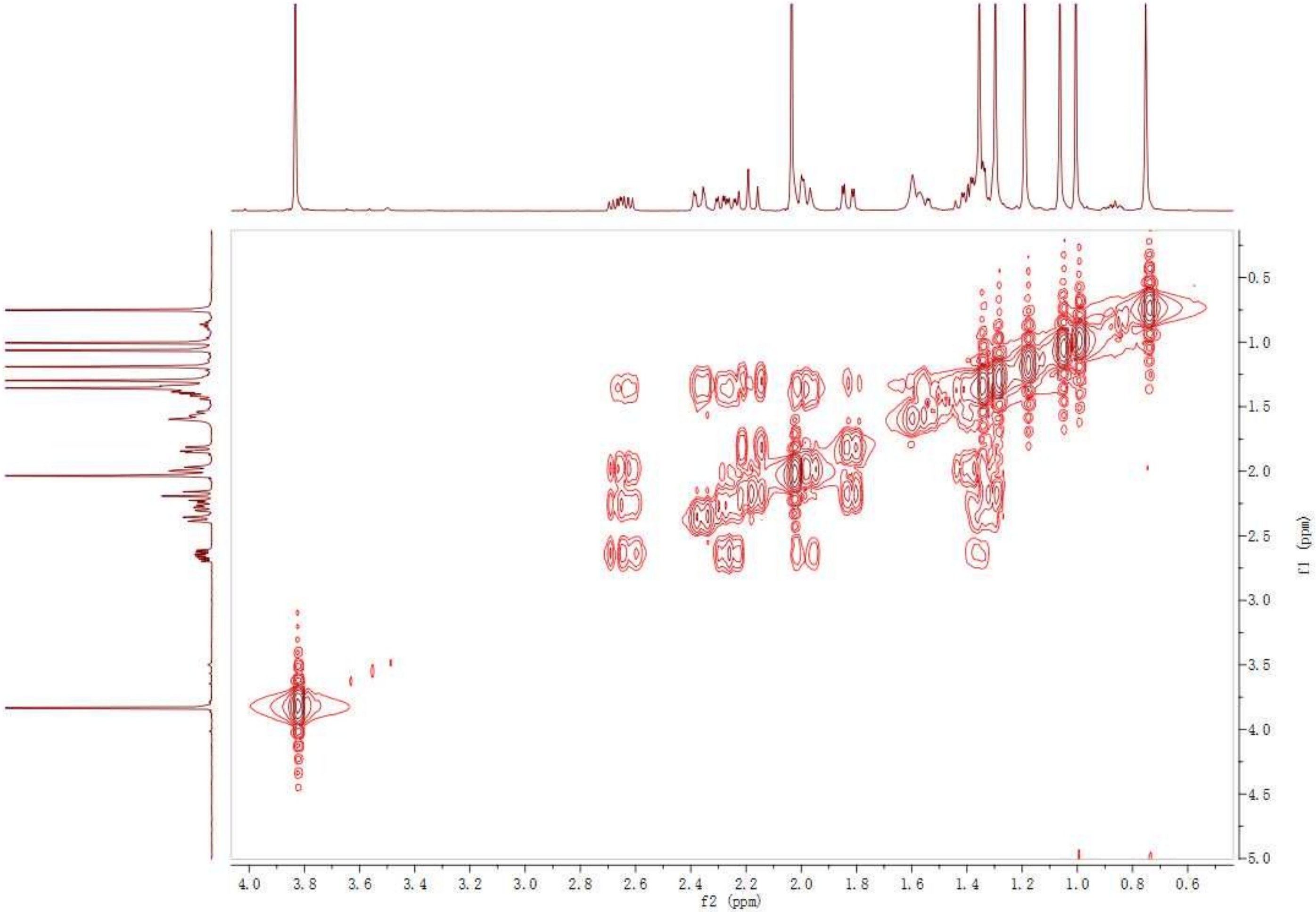

NOESY of compound **1** (in $CHCl_3$)

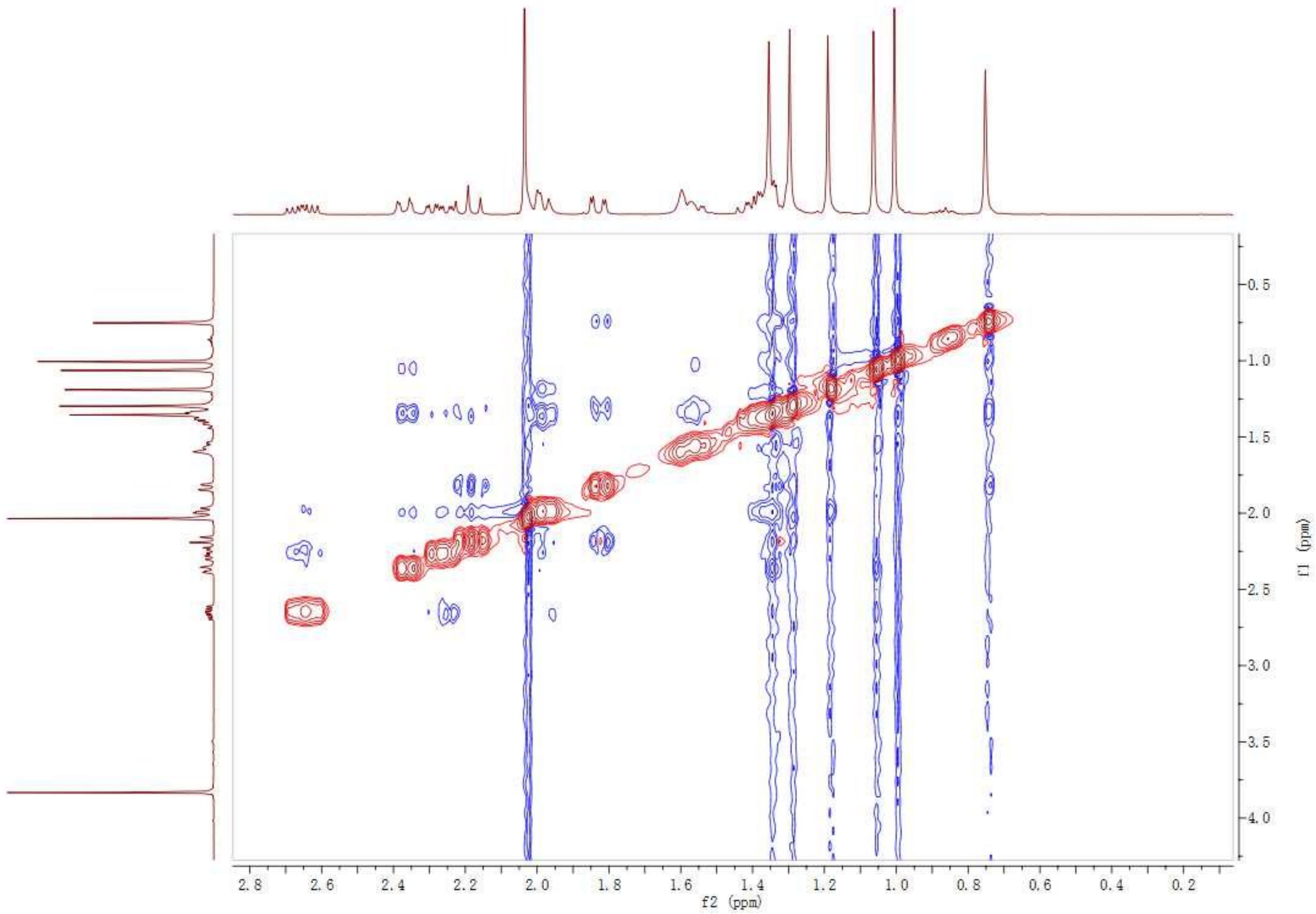

UV of compound **1**

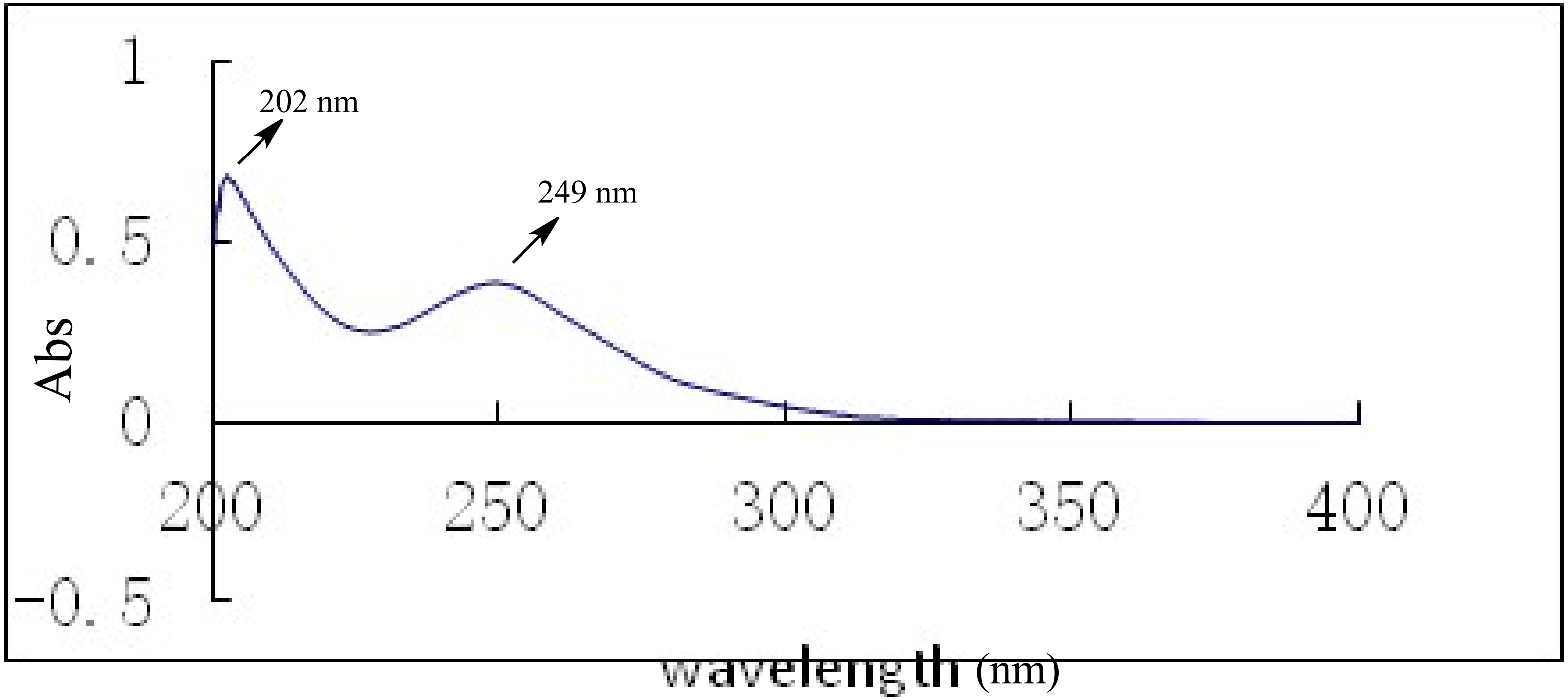

IR of compound **1**

E:\□ □ □ □ □ \□ □ □ \20141229\002.0

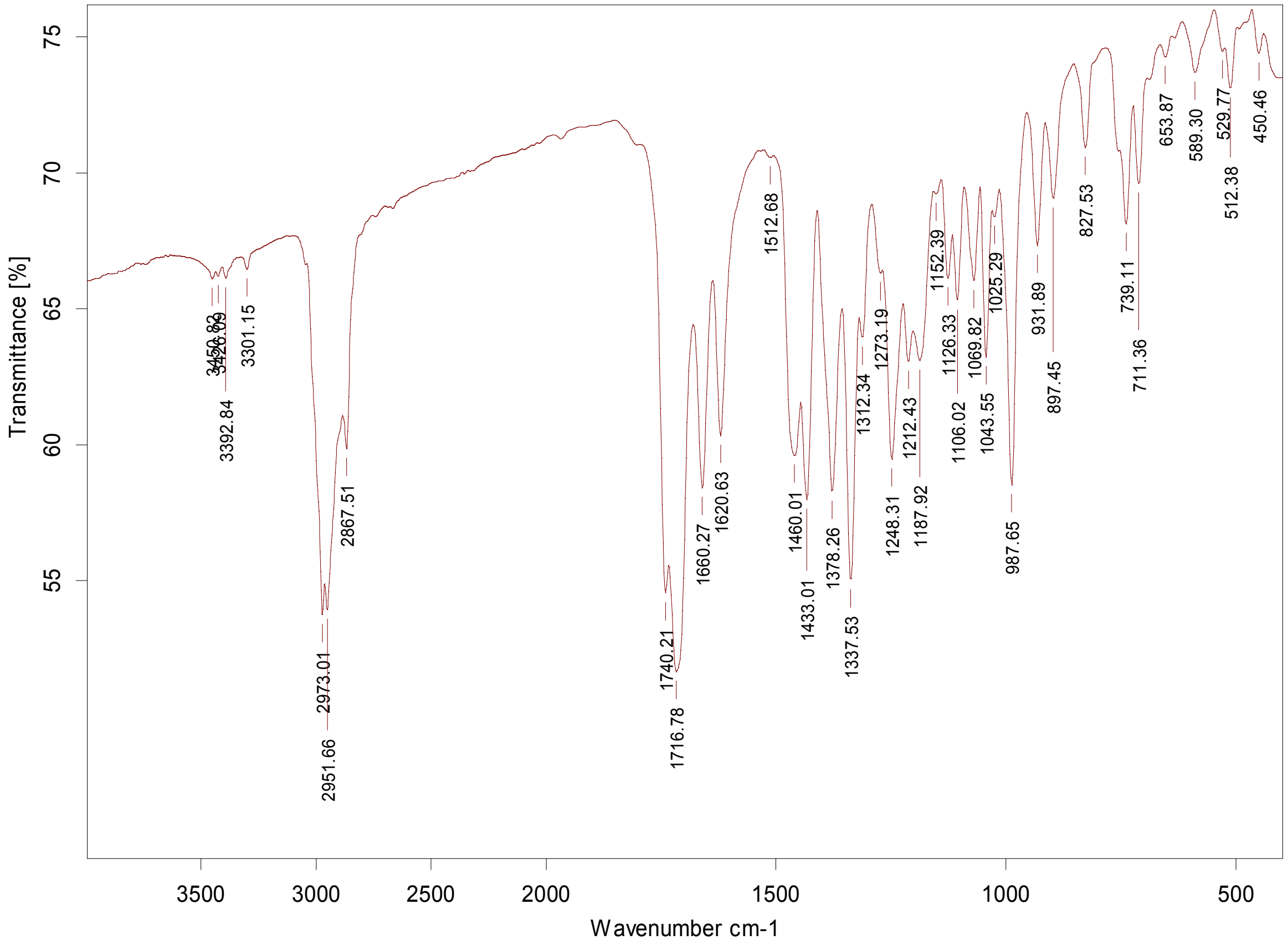

CD of compound **1**

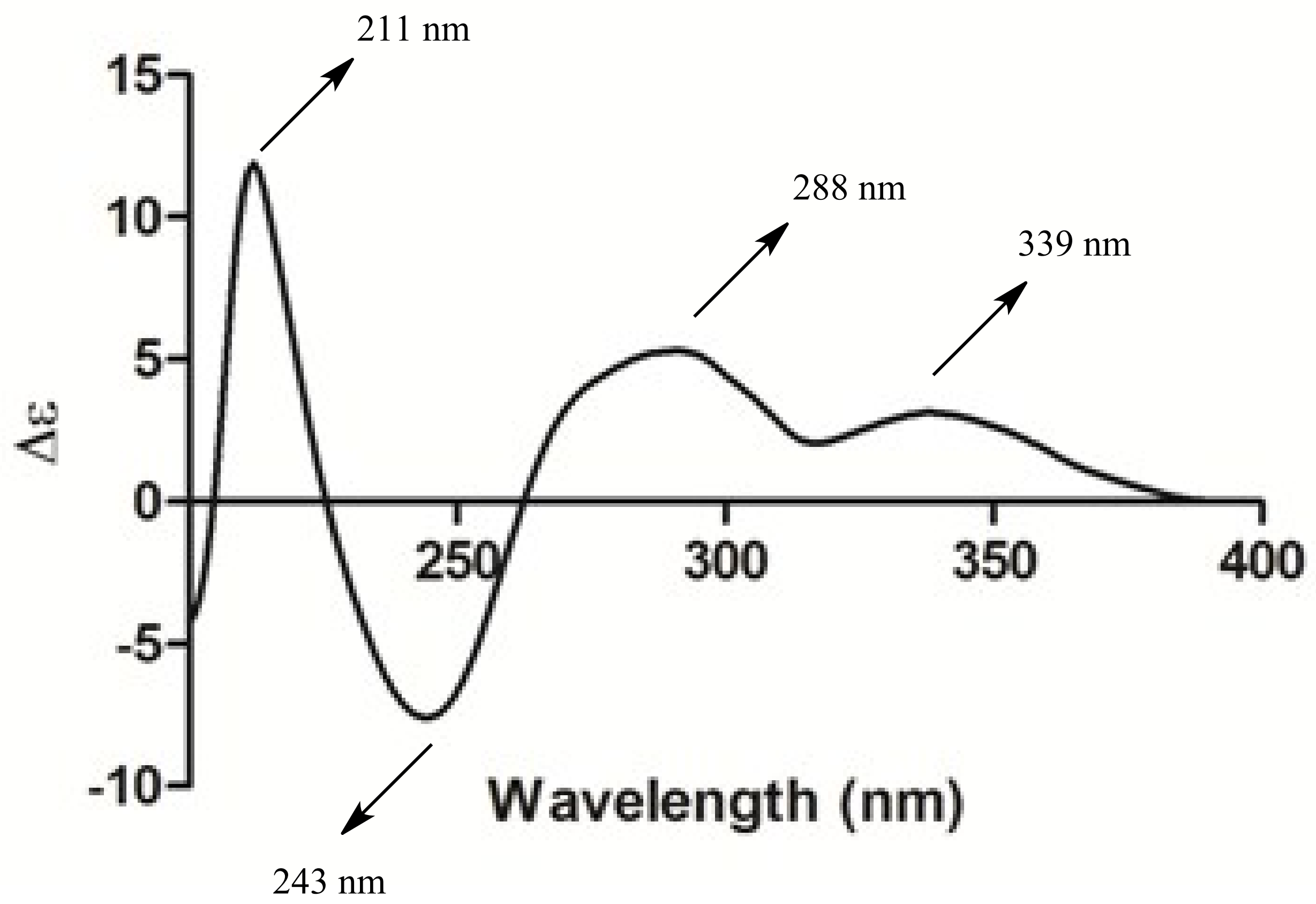

HRESIMS of compound **2**

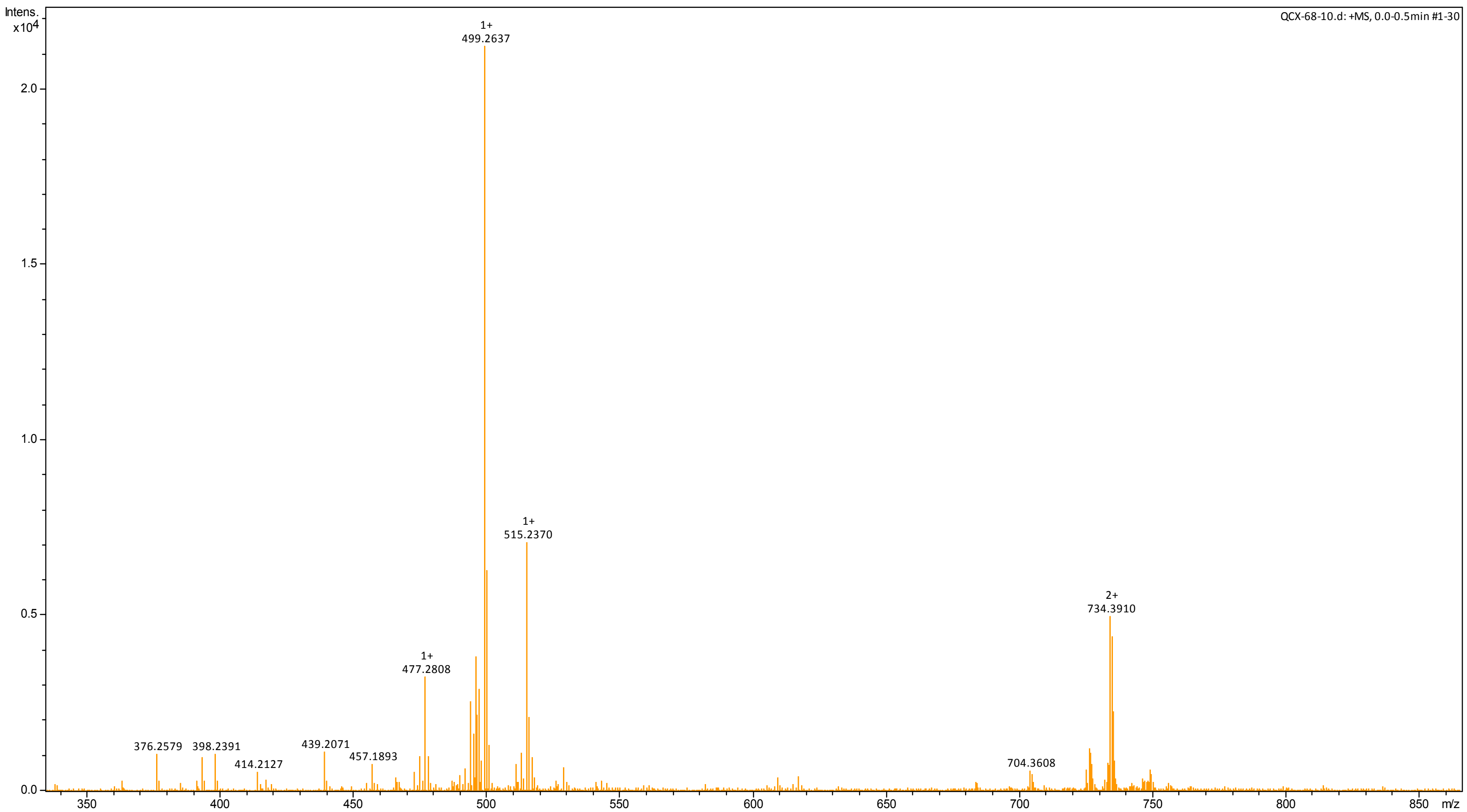

^{1}H NMR of compound **2** (in $CHCl_3$)

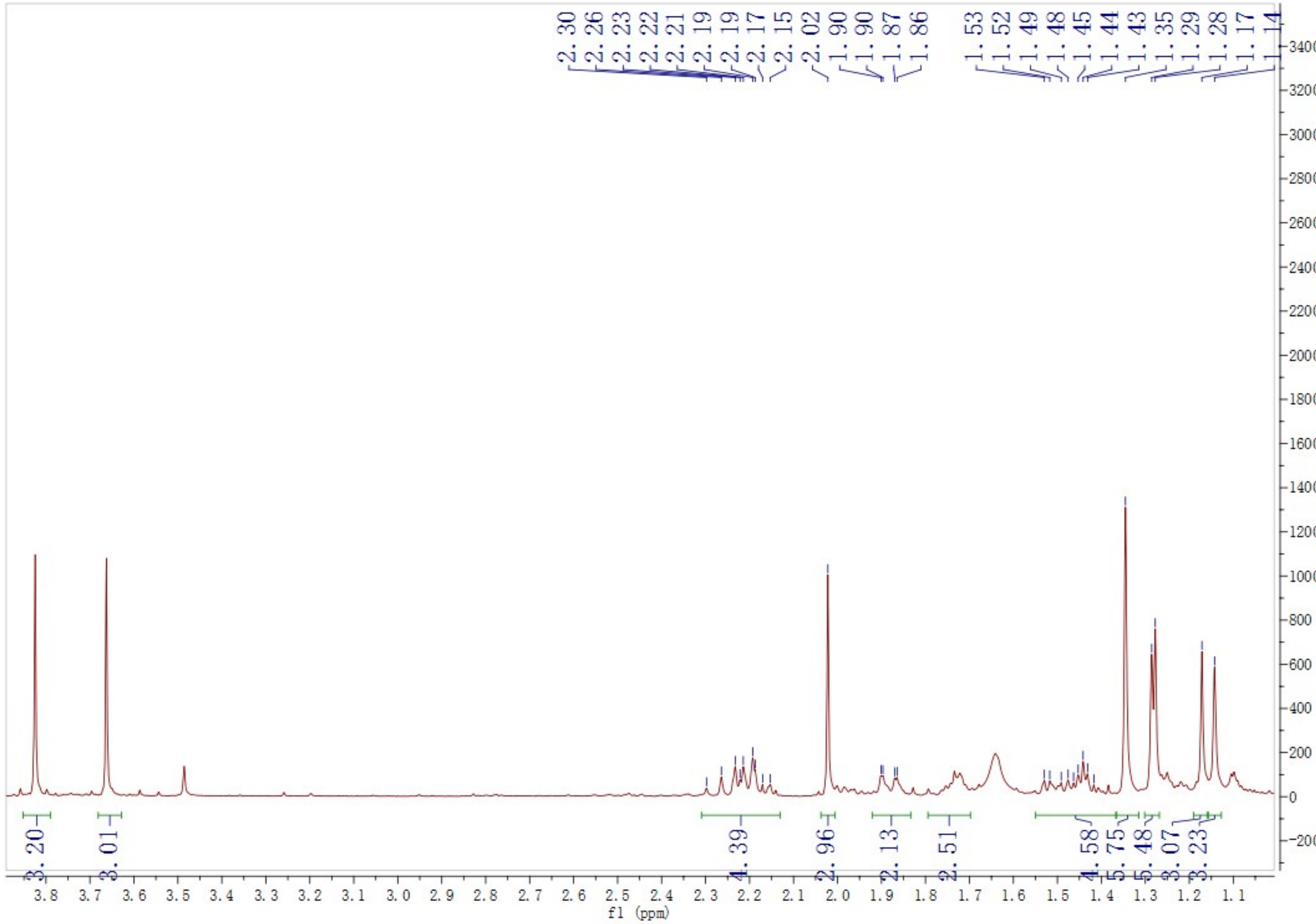

^{13}C NMR of compound **2** (in $CHCl_3$)

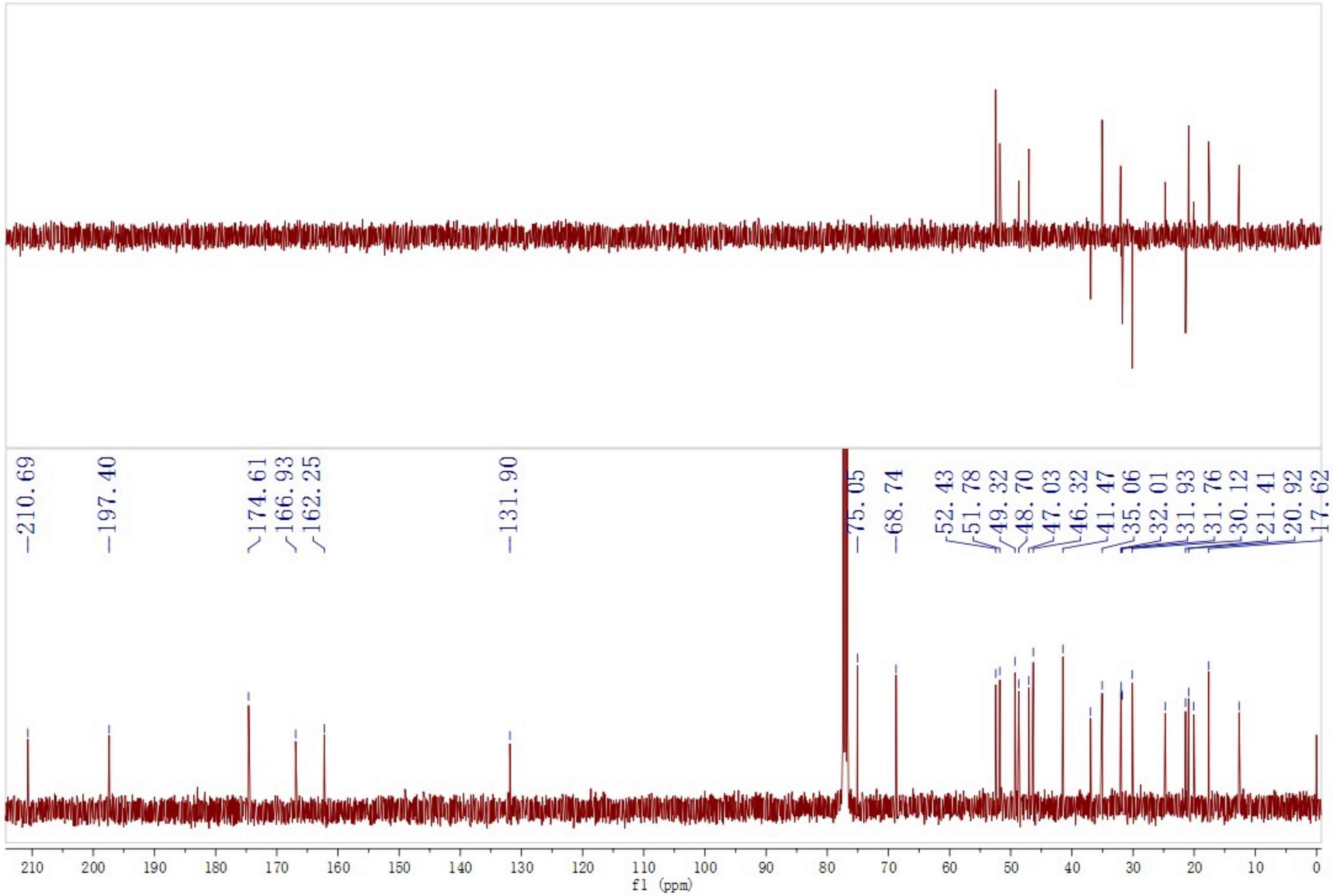

HSQC of compound **2** (in $CHCl_3$)

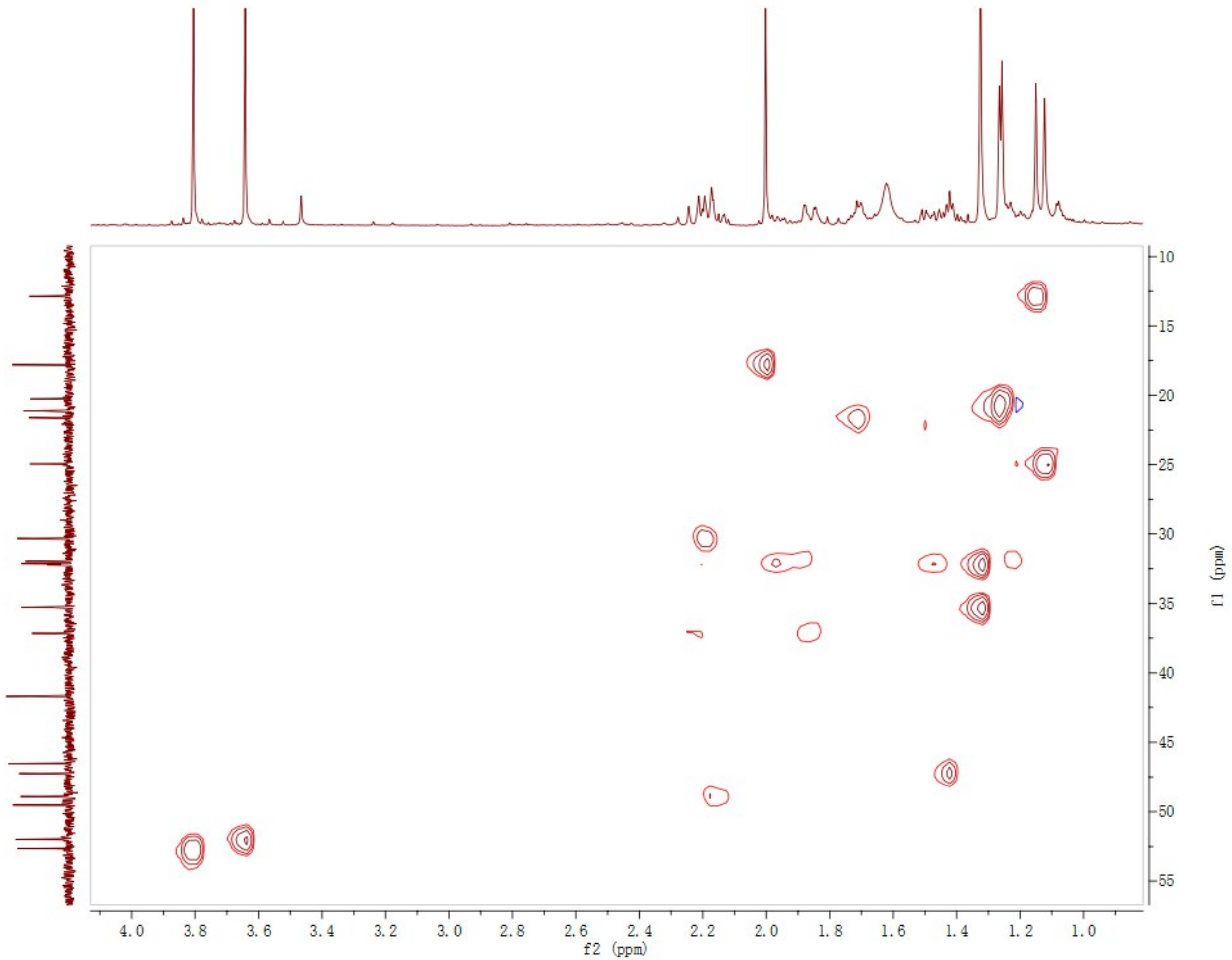

^{1}H–^{1}H COSY of compound **2** (in $CHCl_3$)

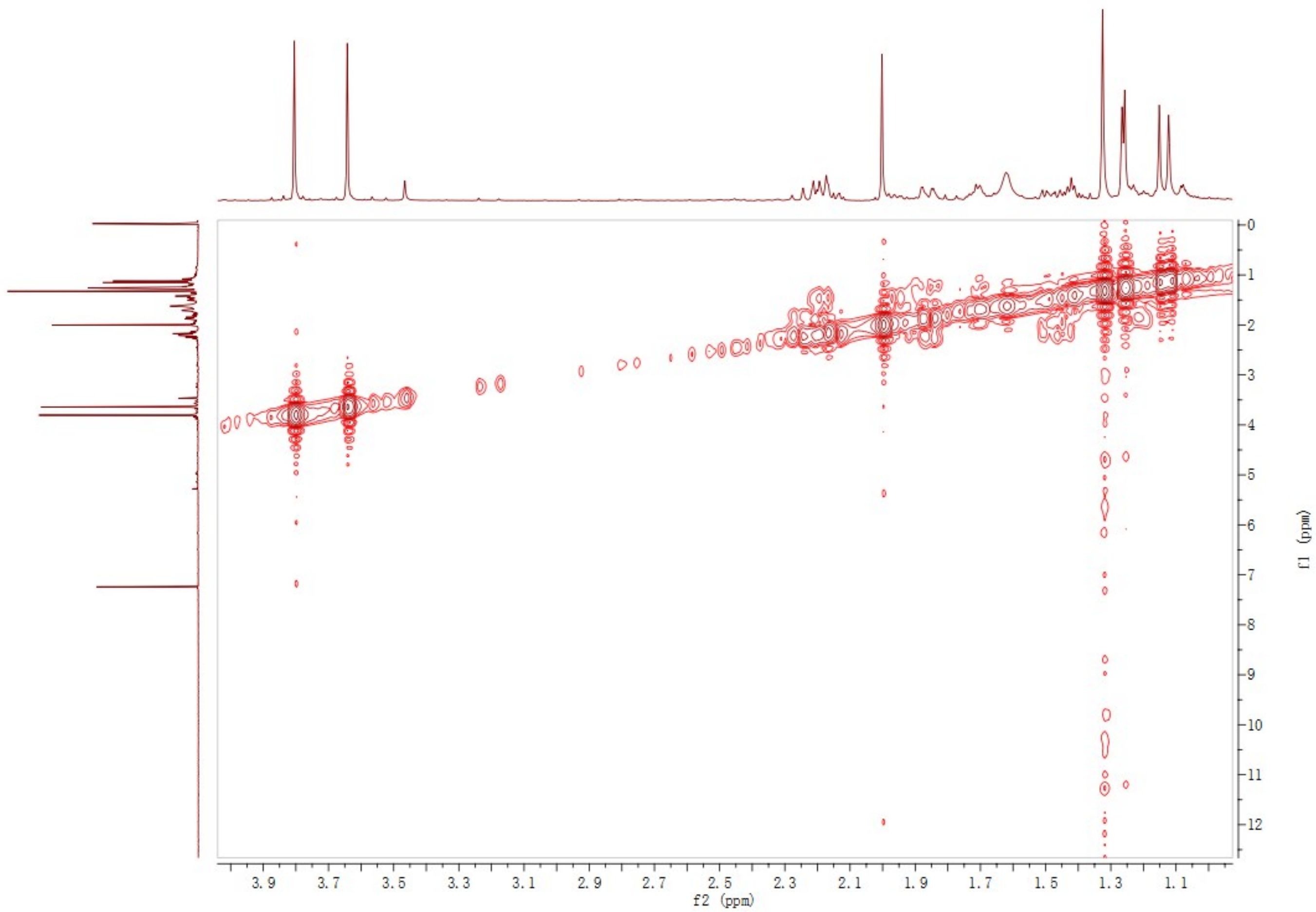

HMBC of compound **2** (in $CHCl_3$)

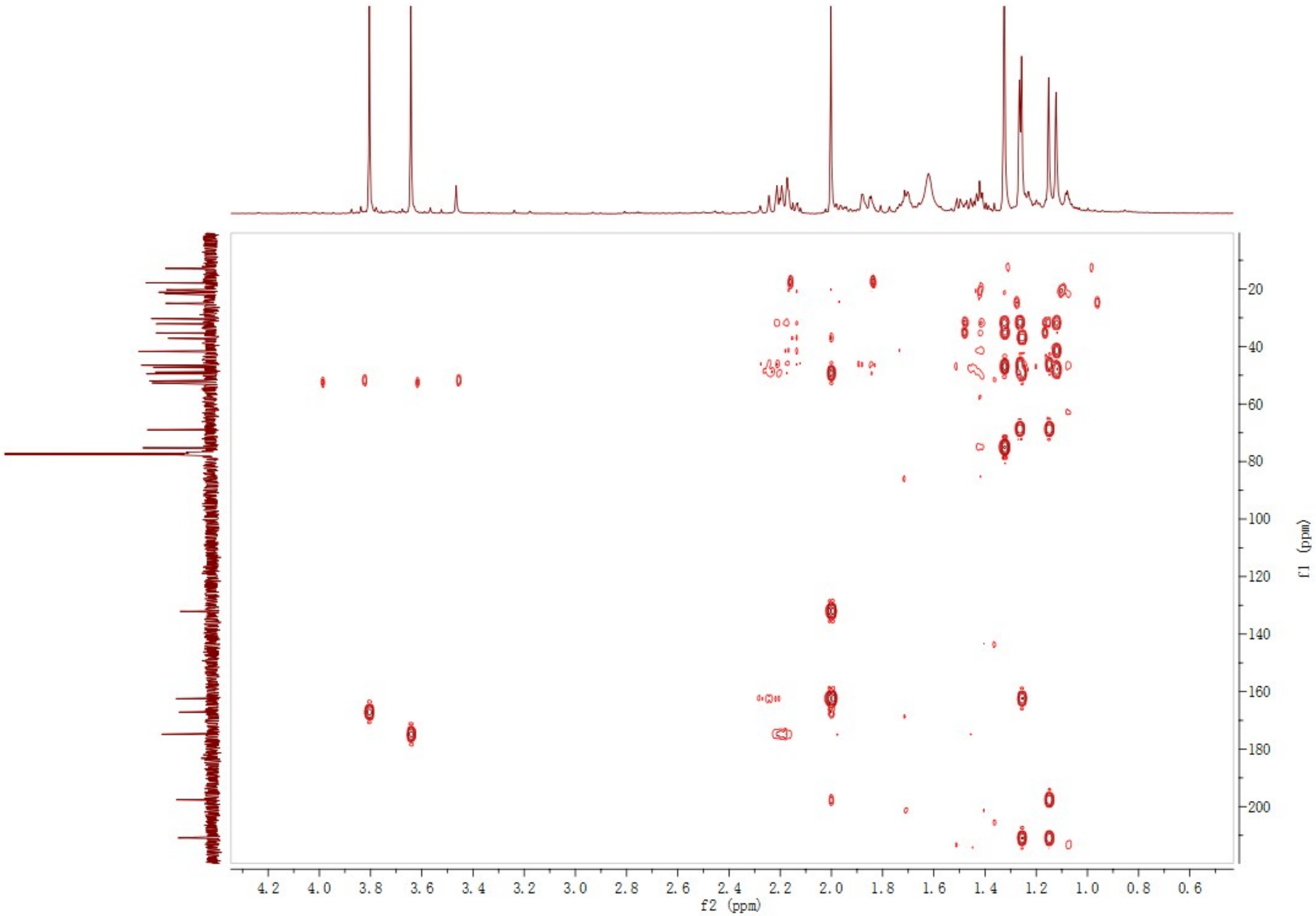

NOESY of compound **2** (in $CHCl_3$)

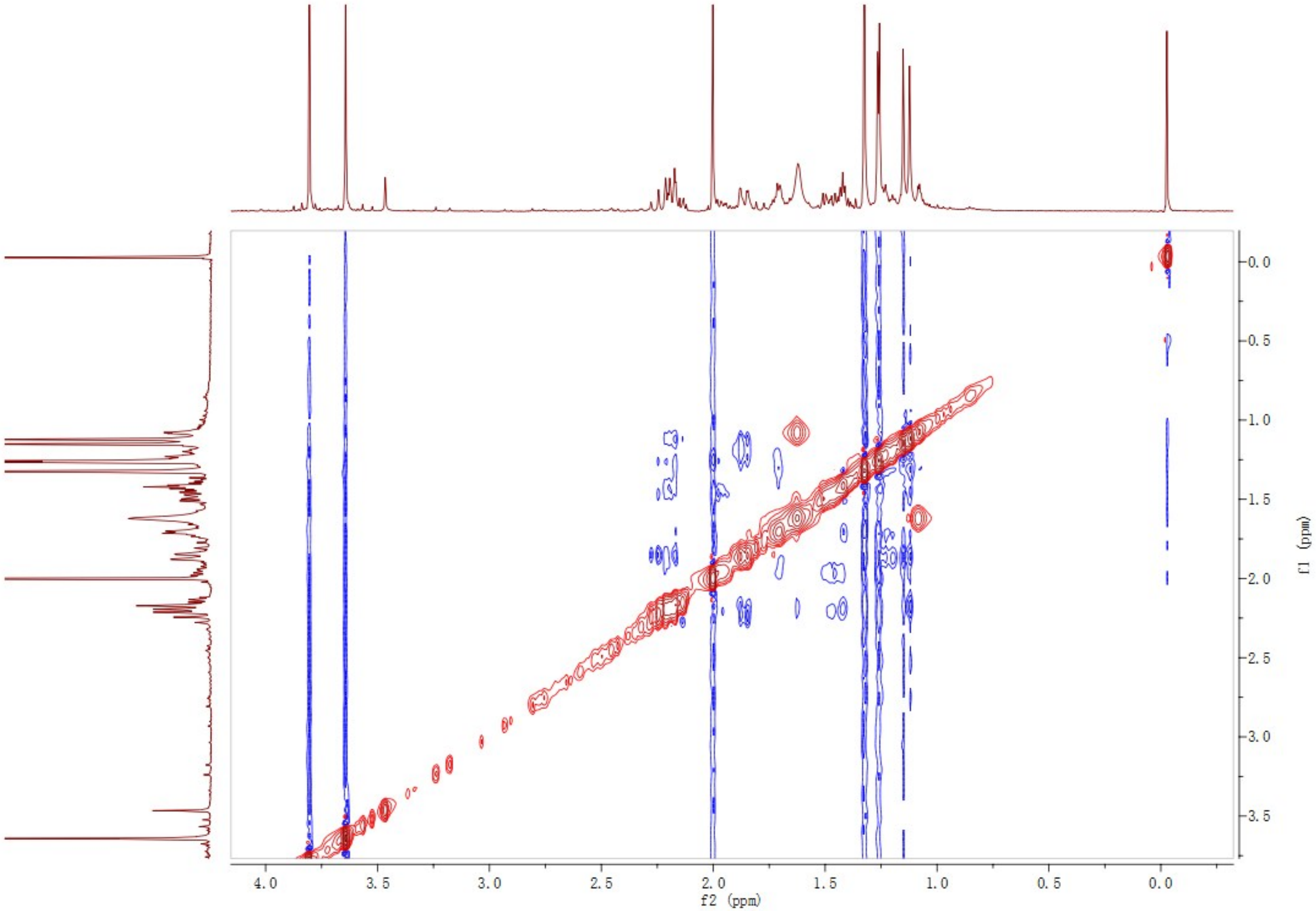

UV of compound **2**

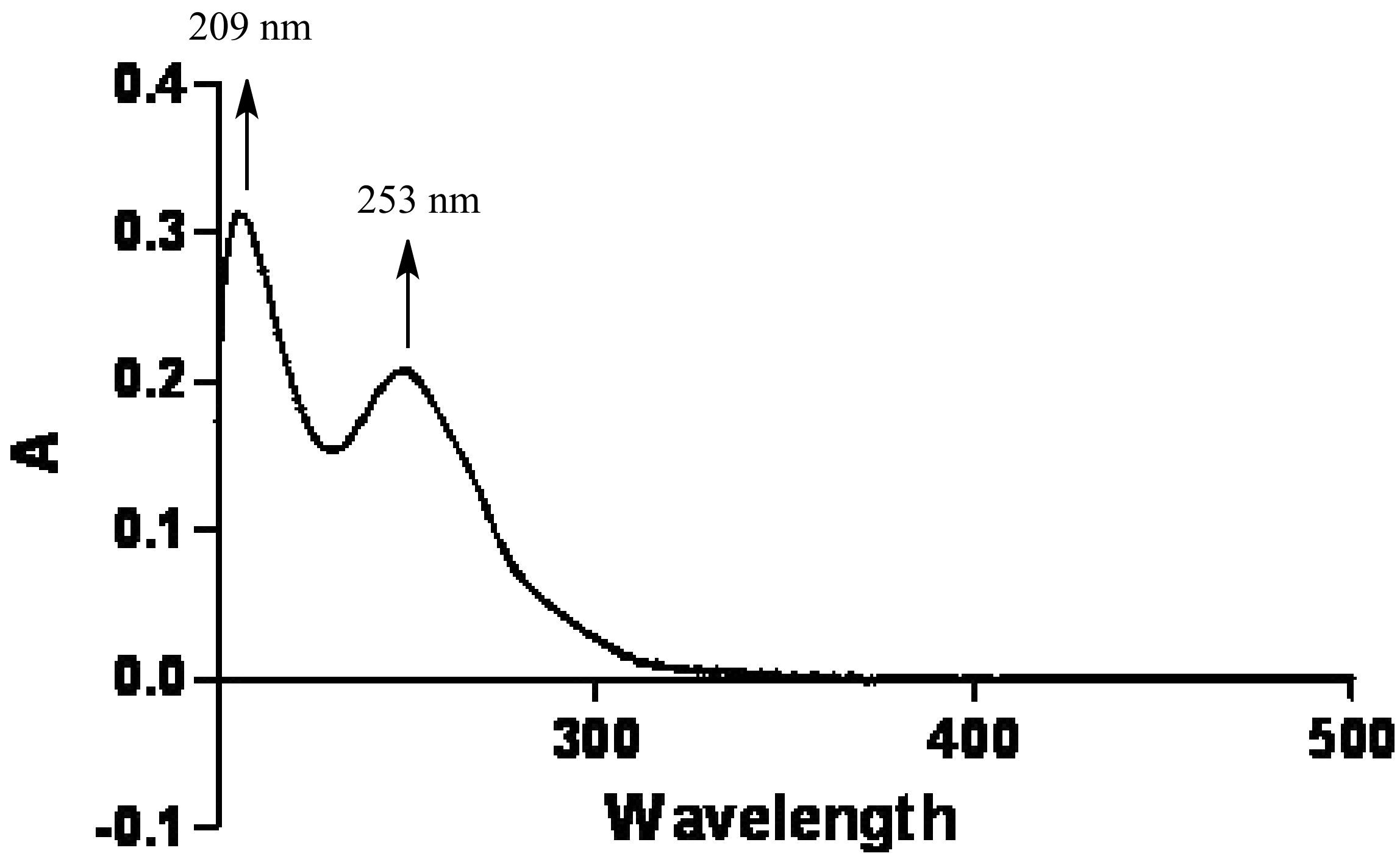

IR of compound **2**

E:\□ □ □ □ □ \□ □ □ \20150320\5-3.0

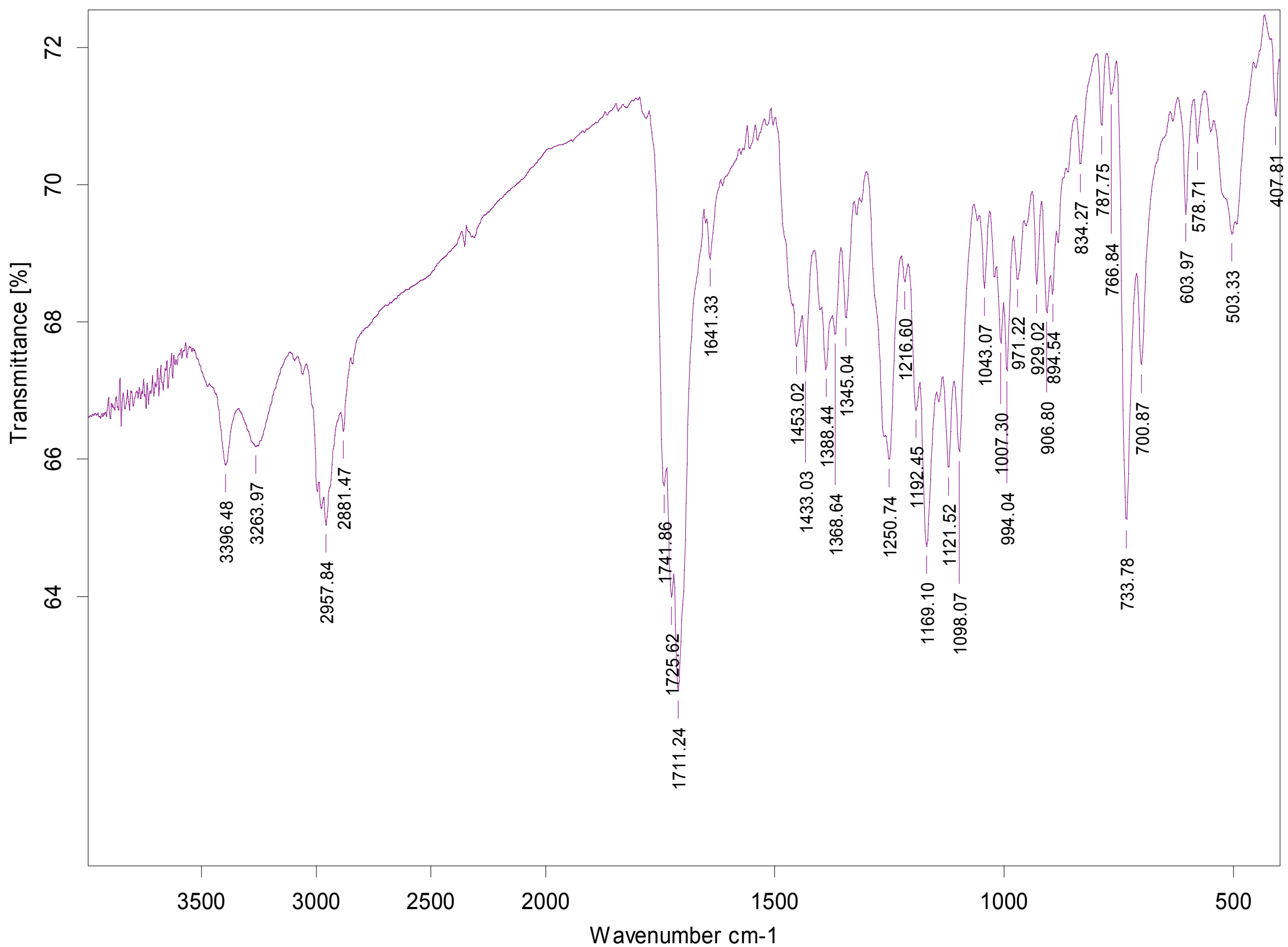

CD of compound **2**

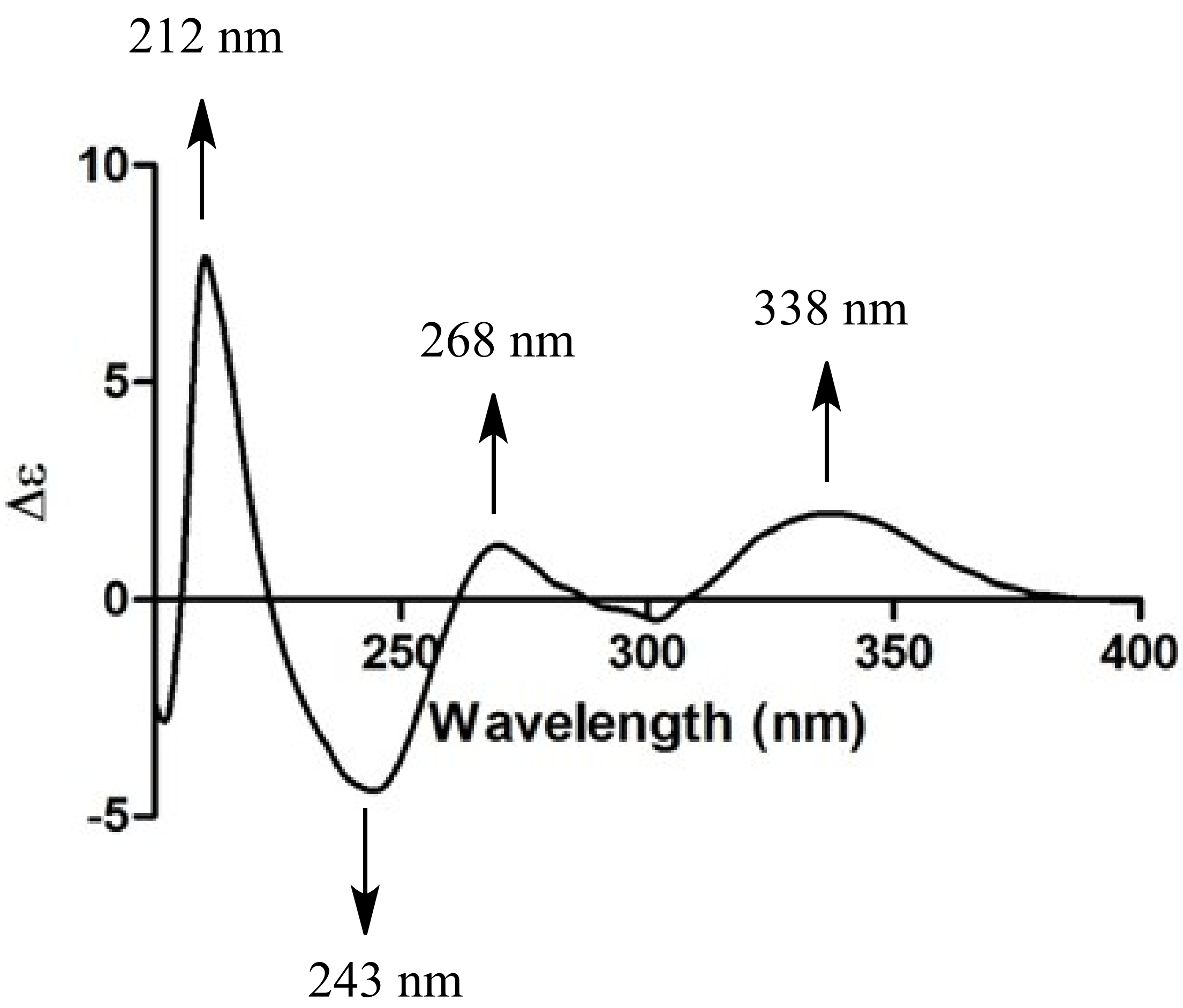

HRESIMS of compound **3**

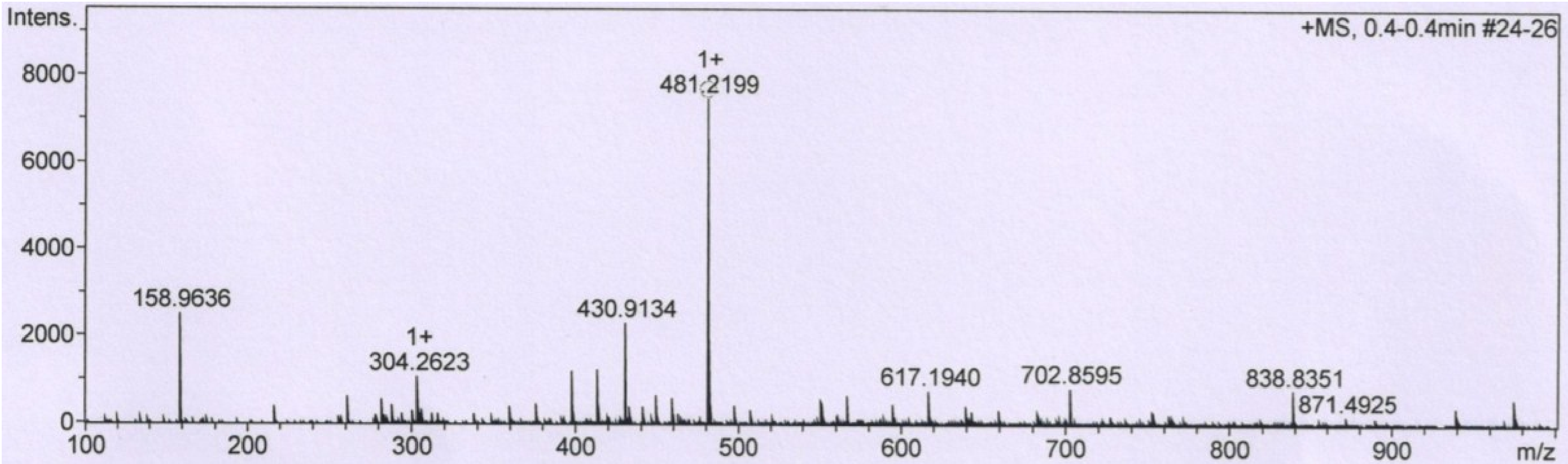
Intens.
+MS, 0.4-0.4min #24-26
8000
6000
4000
2000
0
1+
481.2199
158.9636
430.9134
1+
304.2623
617.1940
702.8595
838.8351
871.4925
100
200
300
400
500
600
700
800
900
m/z

^{1}H NMR of compound **3** (in $CHCl_3$)

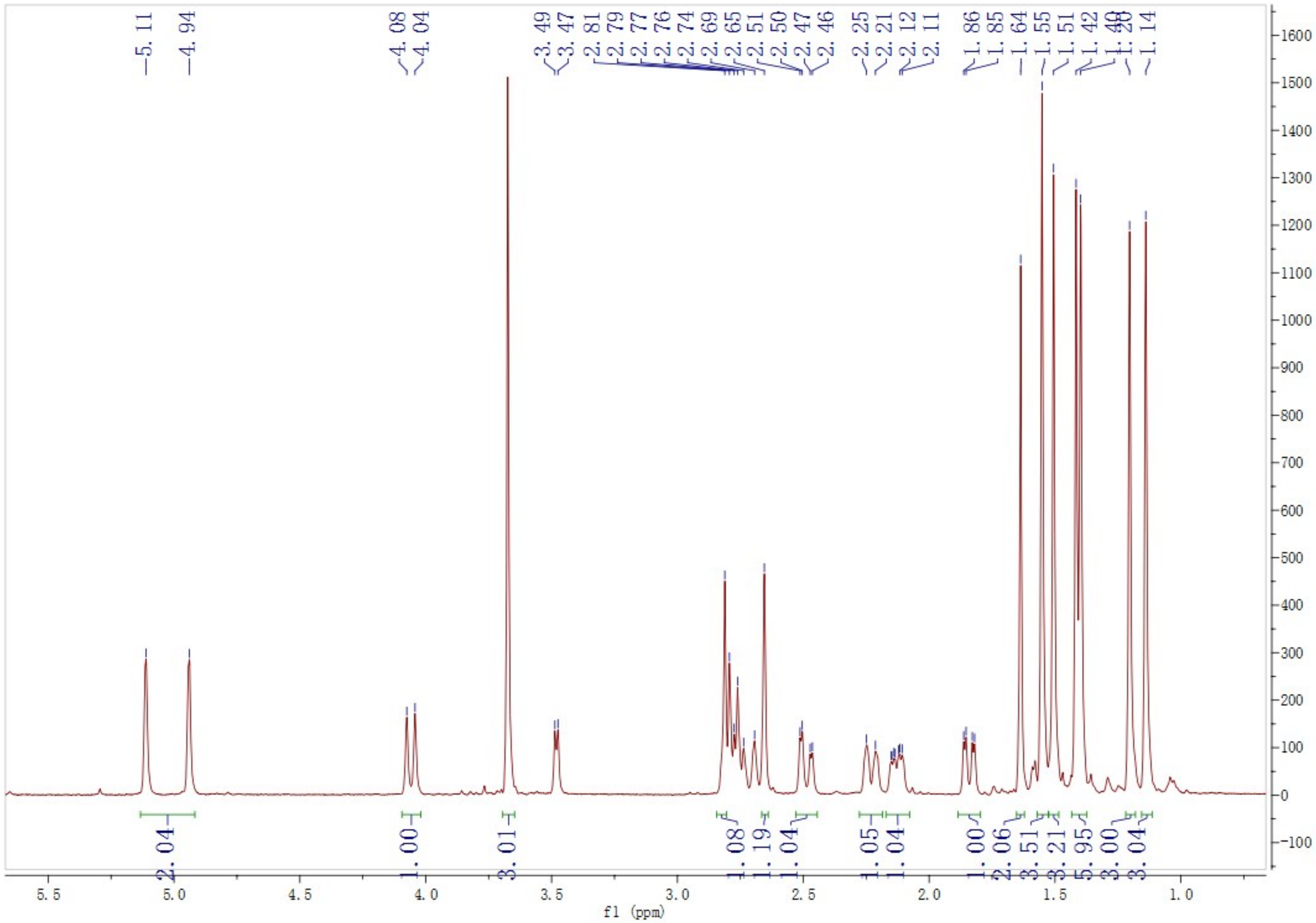

^{13}C NMR of compound **3** (in $CHCl_3$)

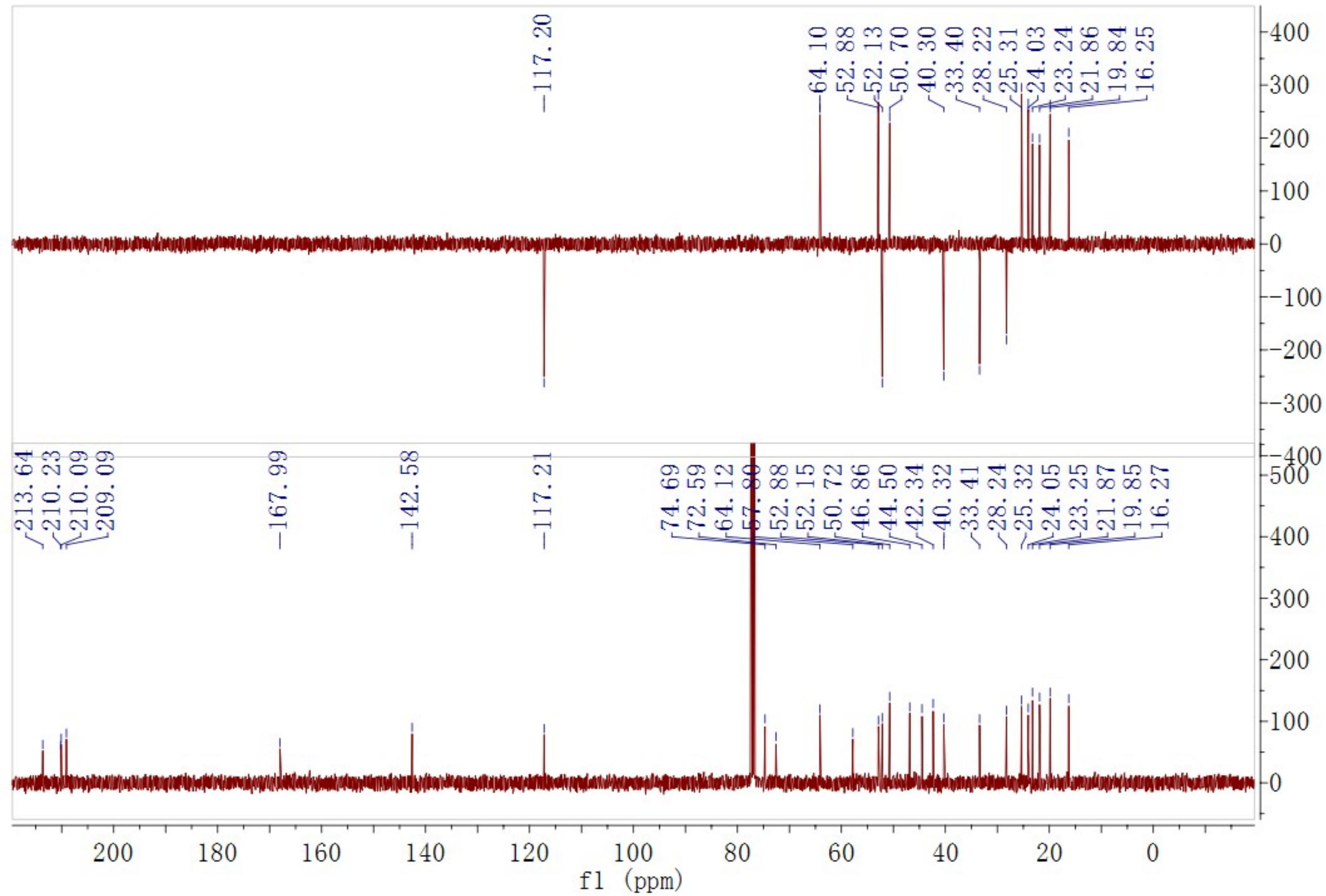

HSQC of compound **3** (in $CHCl_3$)

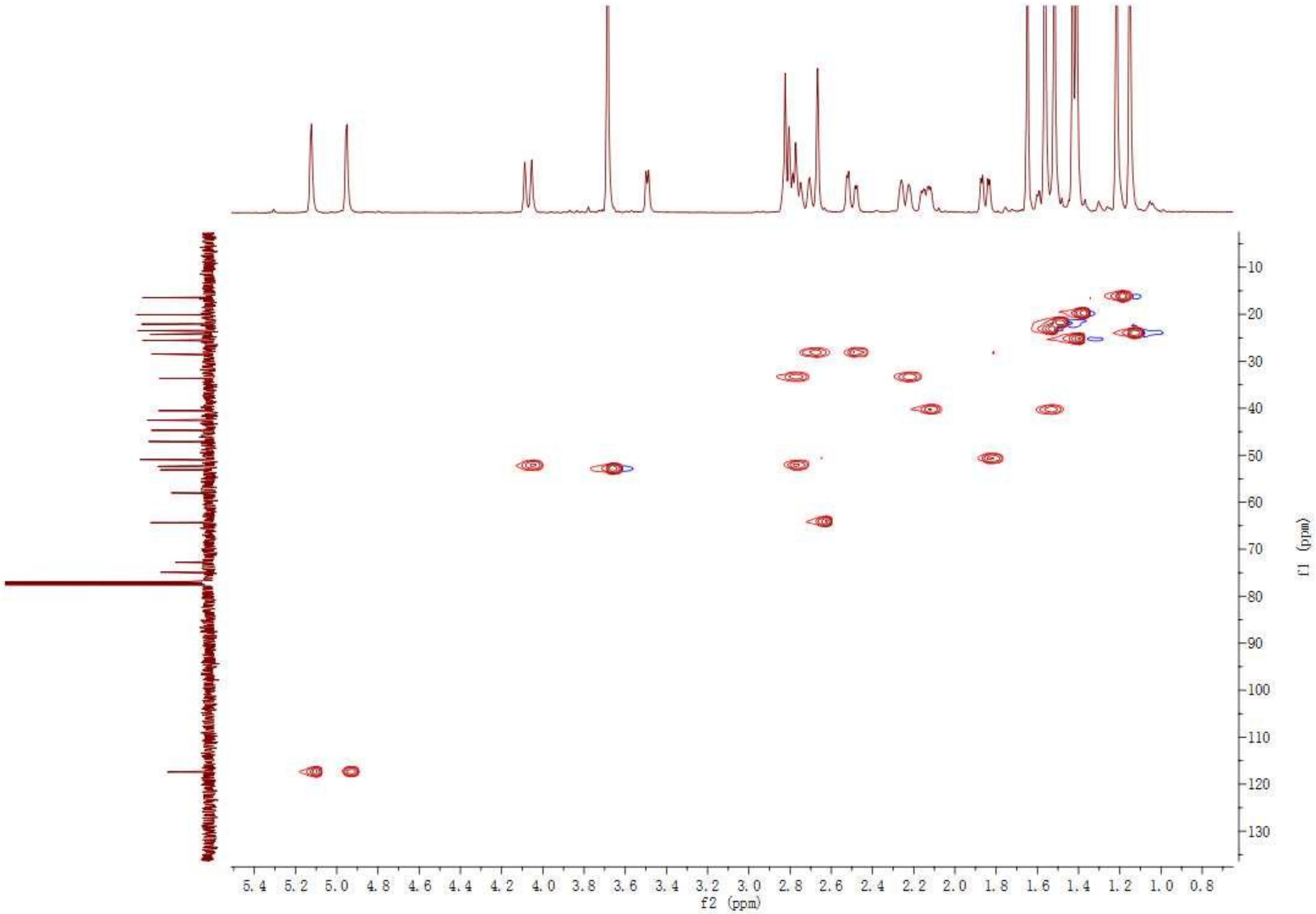

^1H–^1H COSY of compound **3** (in $CHCl_3$)

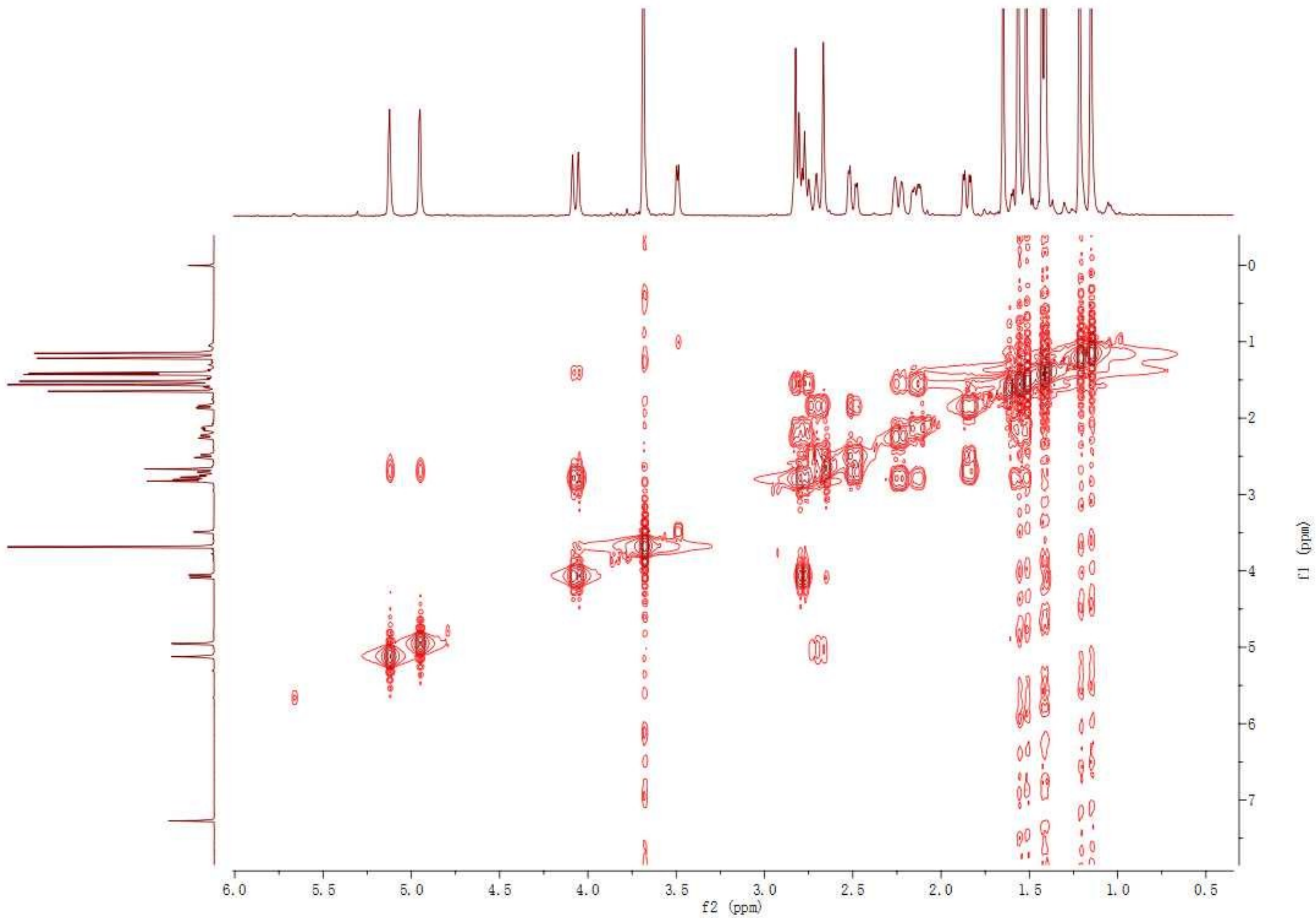

HMBC of compound **3** (in $CHCl_3$)

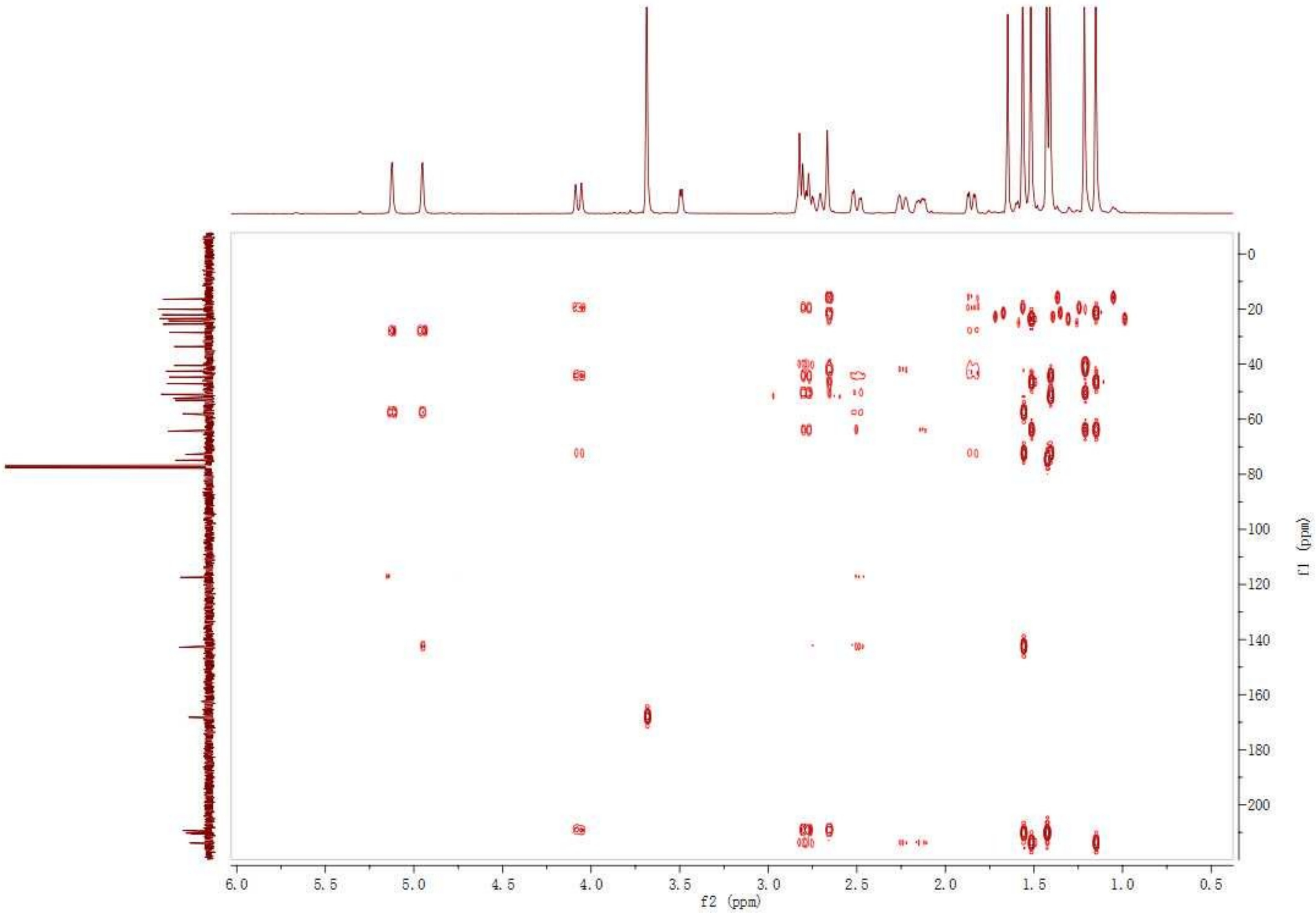

NOESY of compound **3** (in $CHCl_3$)

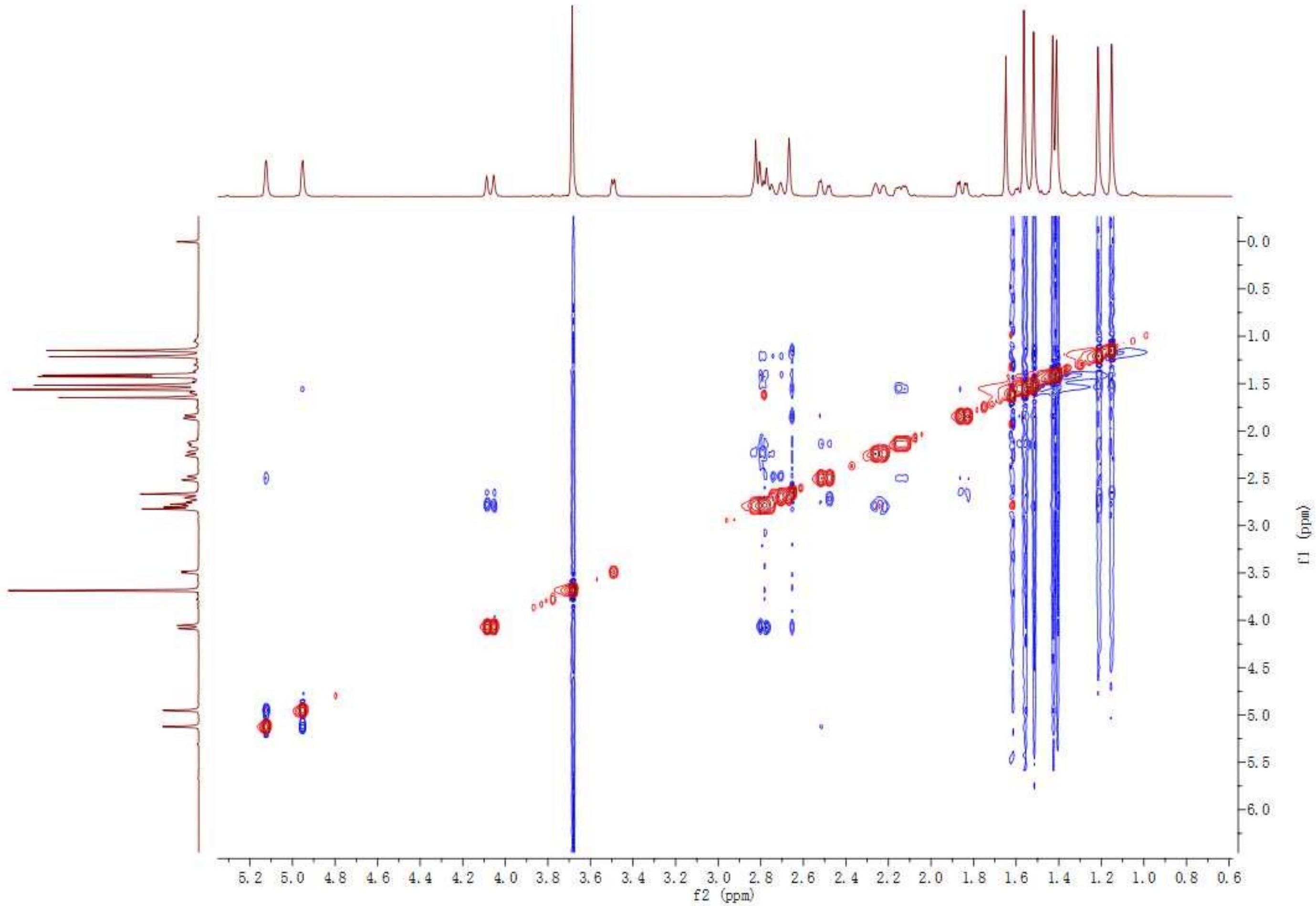

UV of compound **3**

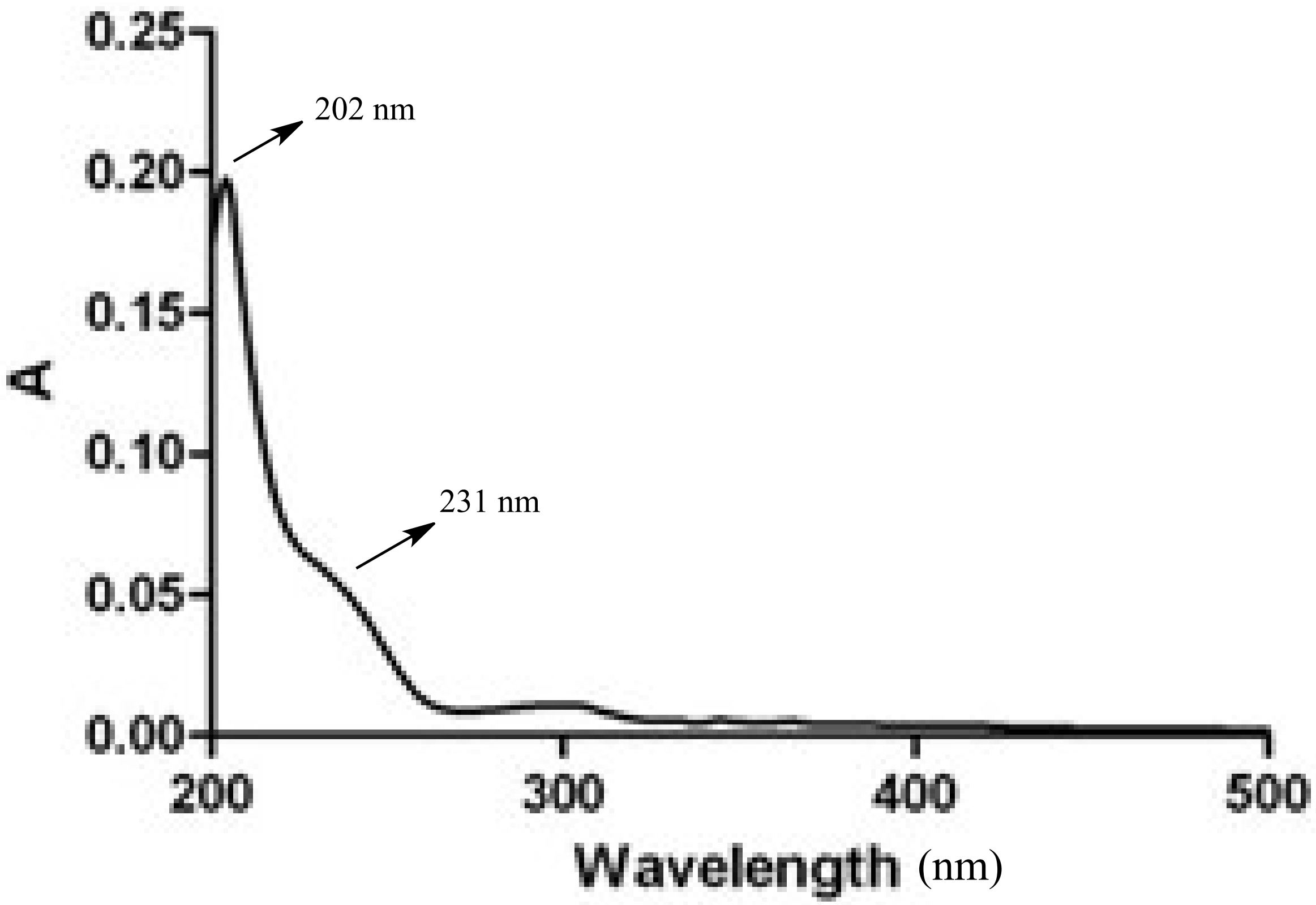

IR of compound **3** (KBrfilm)

E:\□ □ □ □ □ \□ □ □ \20141229\001.0

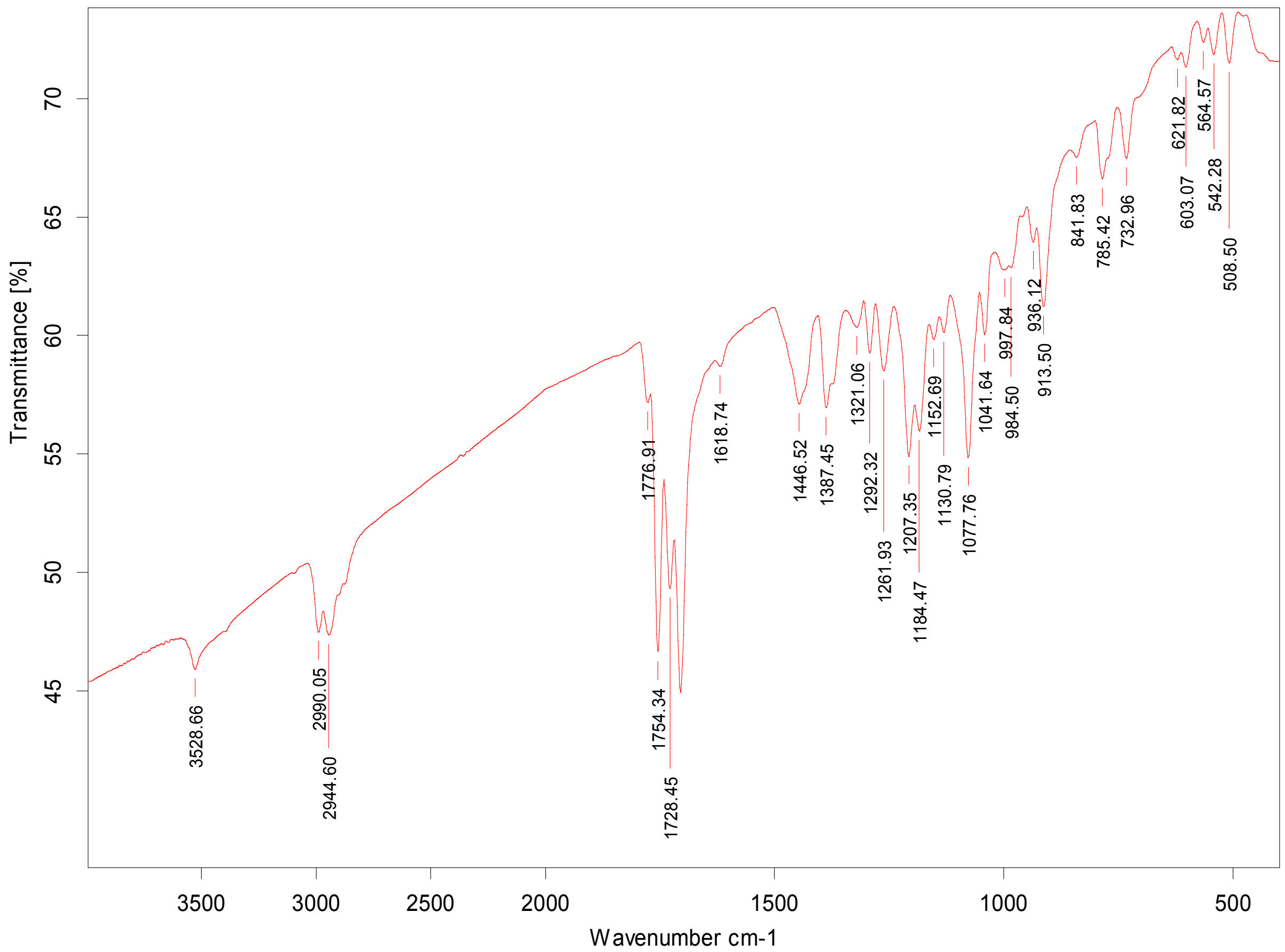

CD of compound **3**

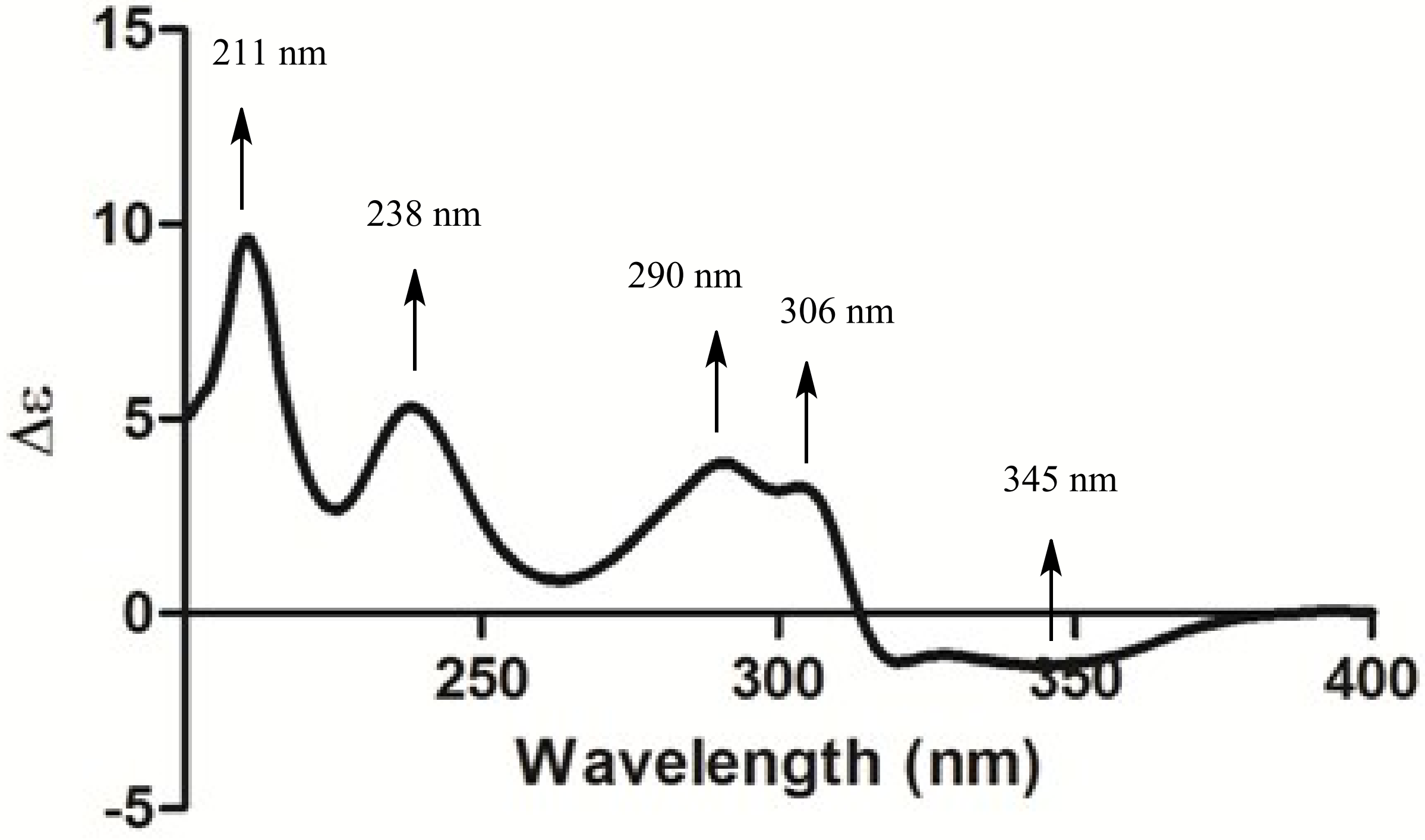

^{1}H NMR of compound **4** (in $CHCl_3$)

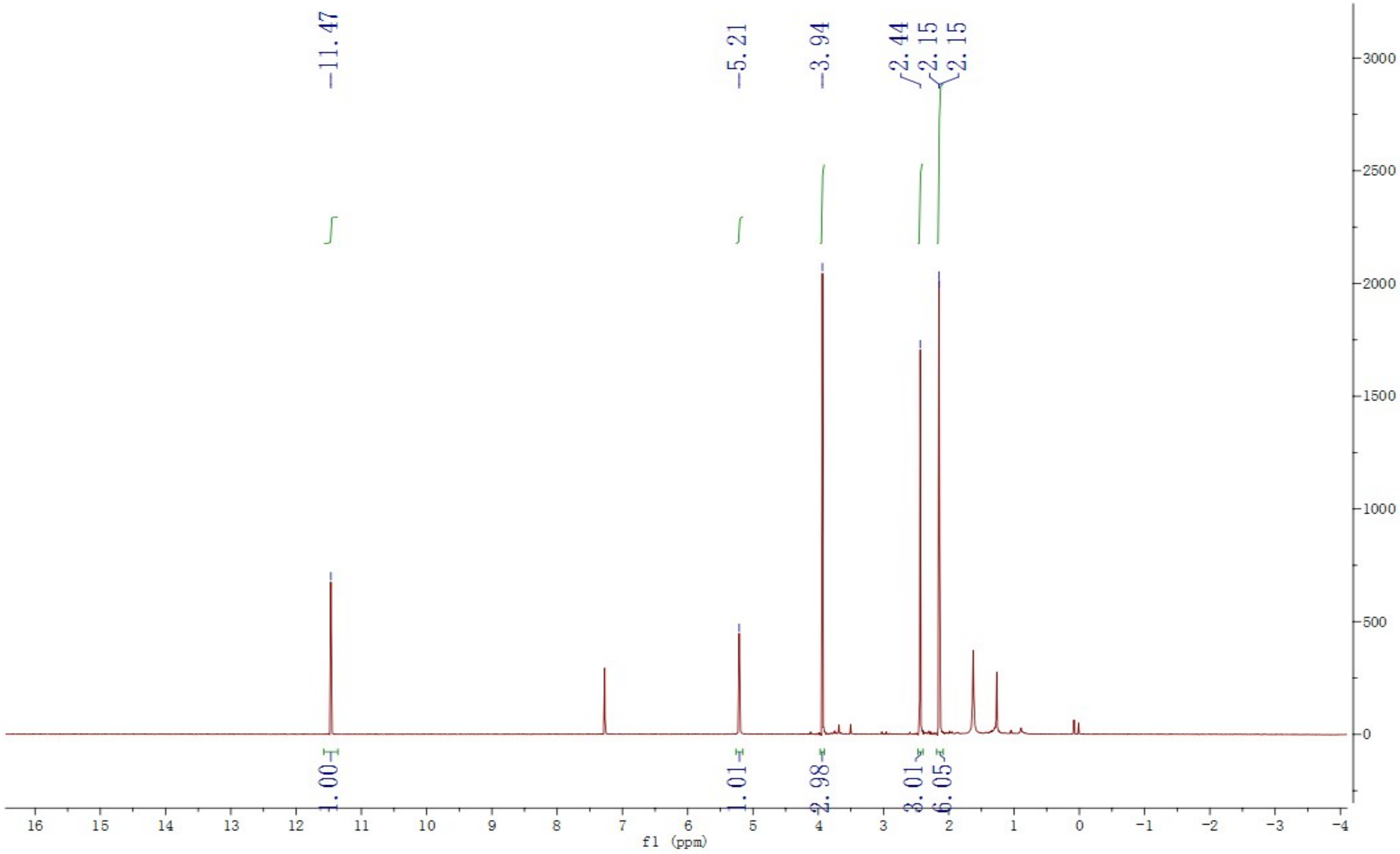

^{13}C NMR of compound **4** (in $CHCl_3$)

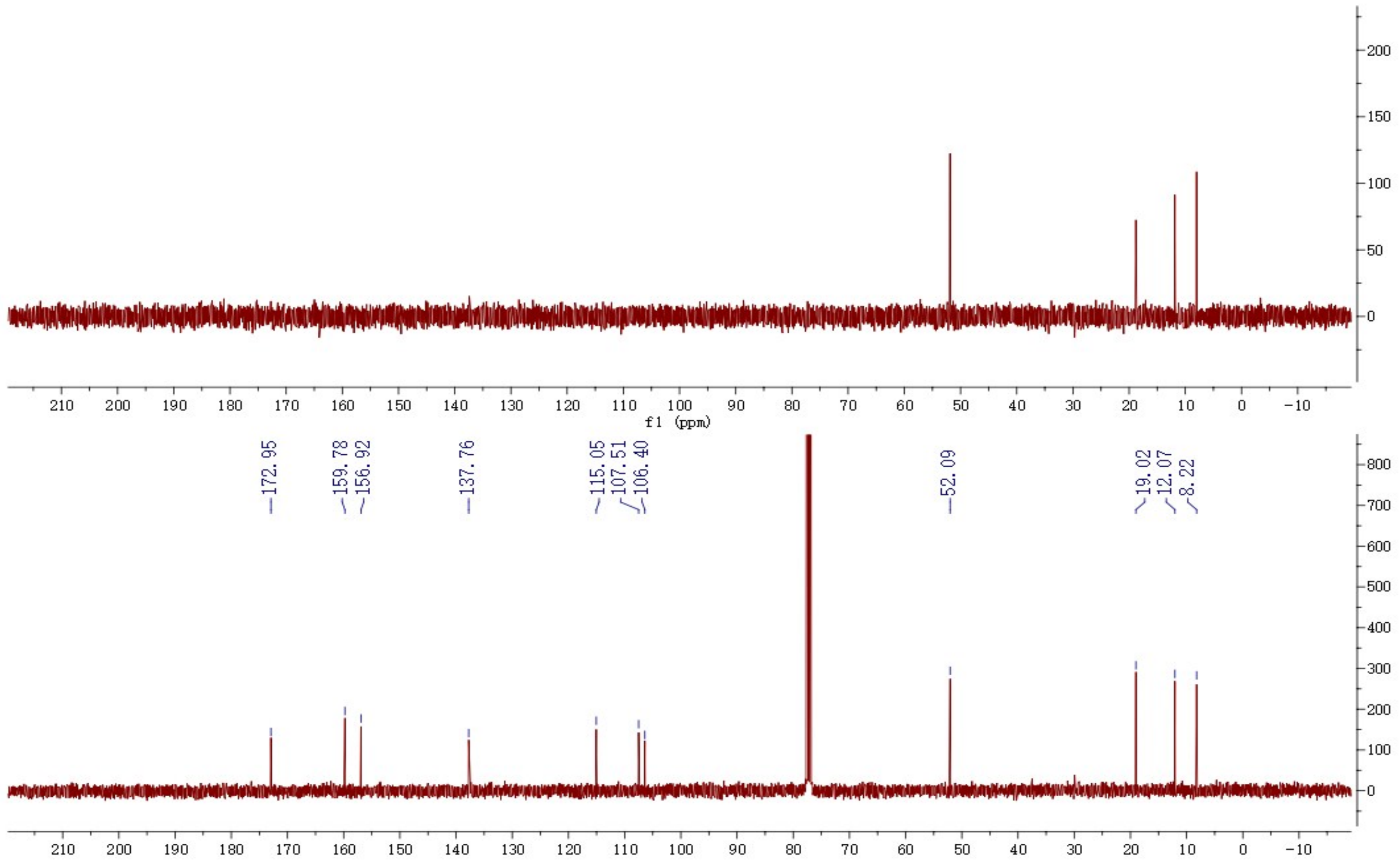
172.95
159.78
156.92
137.76
115.05
107.51
106.40
52.09
19.02
12.07
8.22
f1 (ppm)
